# Identification and Analytical Characterization of a Novel Synthetic Cannabinoid-Type Substance in Herbal Material in Europe

**DOI:** 10.3390/molecules26040793

**Published:** 2021-02-03

**Authors:** Emmanouil D. Tsochatzis, Joao Alberto Lopes, Margaret V. Holland, Fabiano Reniero, Giovanni Palmieri, Claude Guillou

**Affiliations:** 1 European Commission, Joint Research Centre, Via E. Fermi 2749, TP 281, I-21020 Ispra (VA), Italy; Emmanouil.TSOCHATZIS@ec.europa.eu (E.D.T.); Margaret.HOLLAND@ec.europa.eu (M.V.H.); Fabiano.RENIERO@ec.europa.eu (F.R.); 2 Department of Food Science, Aarhus University, Agro Food Park 48, 8200 Aarhus N, Denmark; 3 Agenzia Dogane Monopoli, Direzione Regionale per la Lombardia, Laboratorio e Servizi Chimici, 20138 Milan, Italy; giovanni.palmieri01@agenziadogane.it

**Keywords:** synthetic cannabinoids, 2F-QMPSB, HR-MS/MS, NMR, GC-MS, cheminformatics

## Abstract

The rapid diffusion of new psychoactive substances (NPS) presents unprecedented challenges to both customs authorities and analytical laboratories involved in their detection and characterization. In this study an analytical approach to the identification and structural elucidation of a novel synthetic cannabimimetic, quinolin-8-yl-3-[(4,4-difluoropiperidin-1-yl) sulfonyl]-4-methylbenzoate (2F-QMPSB), detected in seized herbal material, is detailed. An acid precursor 4-methyl-3-(4,4-difluoro-1-piperidinylsulfonyl) benzoic acid (2F-MPSBA), has also been identified in the same seized material. After extraction from the herbal material the synthetic cannabimimetic, also referred to as synthetic cannabinoid receptor agonists or “synthetic cannabinoids”, was characterized using gas chromatography-mass spectrometry (GC-MS), ^1^H, ^13^C, ^19^F and ^15^N nuclear magnetic resonance (NMR) and high-resolution tandem mass spectrometry (HR-MS/MS) combined with chromatographic separation. A cheminformatics platform was used to manage and interpret the analytical data from these techniques.

## 1. Introduction

Recent studies have reported a significant increase in the number of novel psychoactive substances (NPS) within the EU recreational drug market [1,2,3,4,5,6]. Currently synthetic cannabinoids form the largest group of NPS being monitored by the European Monitoring Centre for Drugs and Drug Addiction (EMCDDA), with as many as 160 detected in Europe since 2008 [1]. Synthetic cannabinoids function as high efficacy CB1 and/or CB2 cannabinoid receptor agonists, in contrast with the low to moderate efficacy of tetrahydrocannabinol (THC), the main psychoactive component of cannabis [6,7,8,9,10,11]. 8-quinolinyl-4-methyl-3-(1-piperidinylsulfonyl) benzoate (QMPSB), was detected in Australia between 2011 and 2012 [9] and is reported to be active in the low nanomolar range as a full agonist of the CB1 receptor [12]. The binding affinity of QMPSB, which was evaluated on the basis of [^3^H]-CP-55940 binding to membranes of HEK cells, expressing human CB1 and CB2 receptors, is reported as Ki(CB1) = 3 nM and Ki(CB2) = 4 nM. According to this study, QMPSB behaves as a highly potent, poorly selective, dual CB1 and CB2 agonist [13].

The evolution of the EU market for NPS, and the speed at which new substances are being created, gives cause for concern. The authorities are struggling to keep up with the surge in these substances of abuse. Moreover, their accessibility via the internet, e-shops and social-networking sites, where they are often mislabeled as “bath salts” or “herbal extracts”, plays a major role in their marketing, sale and distribution. An example of this was seen in the seizure of plant material containing QMPSB, which was packaged and labeled as herbal incense [9,10,11,12,13,14,15,16,17,18,19,20]. Many attempts have been made to try to reduce the production of such products, also marketed as potpourri, herbal tea or “Spice”, and all containing synthetic cannabinoids [9,19].

EU customs authorities are responsible for collecting and safeguarding customs duties, as well as controlling the flow of goods into, and out of, the EU, often acting as the first contact point for NPS as they reach Europe via its various borders (maritime, air and terrestrial) [6,21]. The customs laboratories generally perform routine analysis, using techniques such as GC-MS and FT-IR, to identify seized substances, usually relying on matches with results already available in spectral libraries [21]. However, advanced analytical tools such as nuclear magnetic resonance (NMR) and high-resolution liquid-chromatography tandem mass-spectrometry (HR-LC–MS/MS) are often also required for the complete identification of the chemical structure of new substances. In previous studies we have described our integrated workflow, combining data from the standard analytical techniques (GC-MS, FT-IR) used in customs laboratories with further analysis using NMR and HR-MS, performed at the European Commission’s Joint Research Centre (JRC) in Ispra, Italy. This approach enabled the rapid identification of several NPS and contributed to the successive search for information about them [22]. 

The current study presents the application of this workflow to the chemical characterization of the new synthetic cannabinoid 2F-QMPSB (quinolin-8-yl 3-((4,4-difluoropiperidin-1-yl)sulfonyl)-4-methylbenzoate) (Figure S1), which was identified in three different samples of herbal material (hereafter identified as samples 7, 8 and 9) which were found, together with several other synthetic cannabinoids, in a postal parcel seized by customs control authorities (see Section 4.2 Seized herbal material samples). Subsequent to its identification, 2F-QMPSB was formally notified to the EMCDDA on the 9^th^ of January 2019 [23]. Here we also present additional data on the analytical characterisation of 2F-QMPSB which led to the identification of a secondary compound, 2F-MPSBA (3-(4,4-difluoropiperidine-1-sulfonyl)-4-methylbenzoic acid)) (Figure S2), which had not yet been identified at the time of notification to the EMCDDA.

## 2. Results

### 2.1. GC-MS Analysis

**Figure 1:**
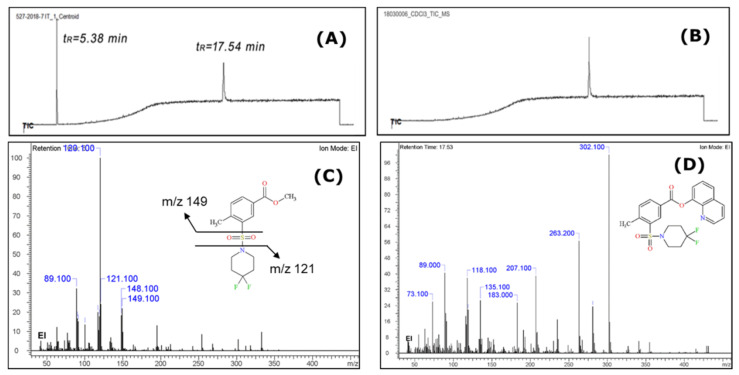
GC total ion chromatogram (Scan mode) of the methanol extract (**A**), chloroform extract (**B**) and EI-MS spectra of the identified methylated precursor of 2F-MPSBA in the methanol extract (t_R_ = 5.38 min) (**C**) and the identified synthetic cannabinoid 2F-QMPSB, (t_R_ = 17.54 min) (**D**).

The qualitative analysis performed using GC-IT-MS (Gas Chromatography-Ion Trap—Mass Spectrometry) of the herbal material methanol extract, carried out by the Italian customs laboratory, revealed two chromatographic peaks at t_R_ = 5.38 min and at t_R_ = 17.54 min. The obtained results were further analyzed with the cheminformatics platform ACD/Spectrus and the obtained chromatogram, along with the resulting mass spectra, are presented in Figure 1. An additional chloroform extract of the same herbal material was performed at the JRC and analysed using GC-MS.

From the GC-IT-MS spectrum analysis of the methanol extract it could be seen that two compounds were present. Through peak picking analysis and interpretation of the mass spectra using the ACD/Spectrus algorithms, it was revealed that the peak at t_R_ =17.54 min refers to the potential synthetic cannabinoid-type substance 2F-QMPSB and the peak at t_R_ = 5.38 min initially looked as if it could be 4,4-difluoropiperidine (*m*/*z* 121). However, after further processing of the results it was confirmed that only the peak at *m*/*z* 120 was correlated with this structure, while there was an abundant fragment at *m*/*z* 149 which was not representative of 4,4-difluoropiperidine. It was found that this substance seemed to be the methylated product of 2F-MPSBA [4-methyl-3-(4,4-difluoro-1-piperidinylsulfonyl) benzoic acid], an acid precursor of 2F-QMPSB (Figure 1C,D). However, this was not identified in the chloroform extract, which suggests that a transesterification reaction occurred during the extraction of the herbal material with methanol (Figure 1A,B). In fact, Blakey et al. already highlighted in their work that these analytical artifacts can occur due to both extraction and/or analysis using GC-MS of synthetic cannabinoids extracted from plant material [9]. These results may indicate the presence of the acid version of that substance in the herbal material that is coextracted with the cannabinoid. Its absence in the GC-MS analysis of the chloroform extract is explained by the analytical column used (DB5-MS), which has a stationary phase that is not suited to the analysis of acids. This can be further explained by the fact that the volatility of this substance is probably lower than that of the cannabinoid itself.

All the identified fragments for both substances, including formulas and mass differences (Da), are presented in Table S1 as produced by the ACD/Labs platform. Using the area normalisation approach, a ratio of 3:7 was estimated for the methylated precursor-to-cannabinoid areas in the GC-IT-MS TIC.

### 2.2. NMR Analysis

**Figure 2:**
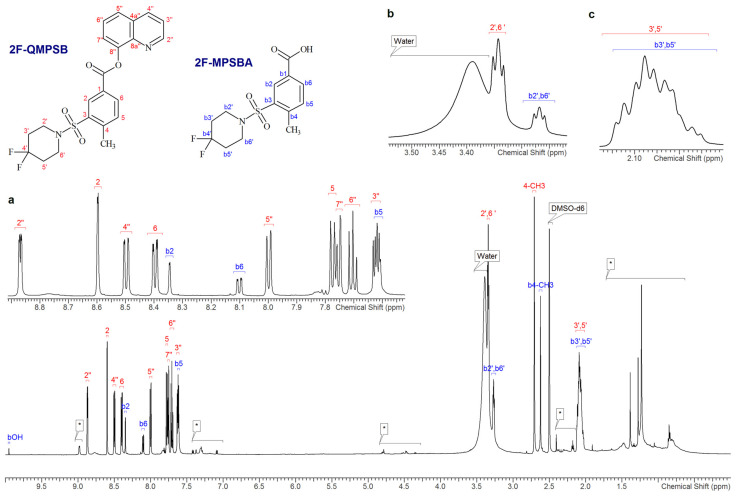
^1^H NMR spectrum of sample 9 in DMSO-d_6_ with expansions of (**a**) the aromatic region (**b**) and (**c**) the aliphatic region. The signals annotated with an asterisk (*) are from other unidentified species extracted from the matrix.

The NMR spectra recorded for the CDCl_3_ extracts showed broad signals at between 0 and 1.8 ppm, which can be attributed to the lipidic fraction extracted from the herbal material (Figure S5). The NMR spectra of the three samples, 7, 8 and 9, also showed similar patterns of signals corresponding to aliphatic chains and aromatic protons whose relative proportions seemed to indicate the presence of two major compounds (Figure S6). For the three samples, the ^19^F NMR spectra also showed a signal indicating the presence of fluorine in the unknown substances investigated (Figure S7). Initially the chemical shifts and overlaps observed for the signals in the aromatic region made it rather difficult to achieve a clear and full interpretation of the spectra in CDCl_3_. However, the spectra obtained for the same extracts, redissolved in DMSO-d_6_, showed a better resolution of the signals, in particular in the aromatic region (See Figure 2, Figures S8 and S9). In contrast with the spectra in CDCl_3,_ the chemical shifts observed for the major signals remain almost constant for all three samples independently of the relative proportions of the two main components (Figure S9).

**Figure 3:**
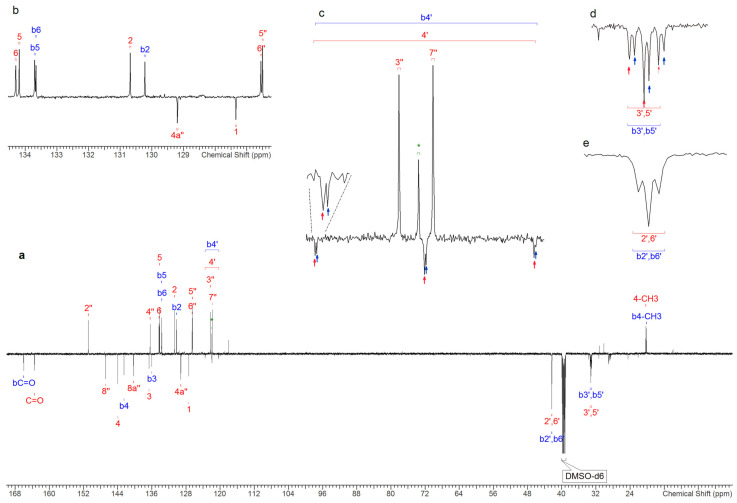
(**a**) ^13^C APT NMR spectrum of 2F-QMPSB in DMSO-d_6_ (sample 7); (**b**) expansion of a part of the aromatic region; (**c**) expansions showing triplets ^1^*J*_C-F_ = 241 Hz from carbons 4’ and b4’ of the two identified compounds, the peak (**δ** = 122 ppm) assigned with an asterisk (*) is from an unidentified secondary species; (**d**) triplets ^2^*J*_C-F_ = 23.2 Hz of 3′,5′ and b3′,b5′ and (**e**) overlapping triplets ^3^*J*_C-F_ = 5.5 Hz for 2’,6’ and b2’,b6’.

The ^1^H NMR spectrum showing 2F-QMPSB and 2F-MPSBA, is presented, with particular focus on the "aromatic region’’ of the spectrum, in Figure 2. The ^1^H NMR, ^13^C and ^3^J_HH_ coupling constants (Table 1) for the identified compounds are consistent with those reported by Blakey, et al. [9]. More specifically, the chemical shifts observed for the piperidinyl unit, in both the ^1^H and the ^13^C NMR spectra (Figure 3), are in accordance with those from both Blakey, et al. and Uchiyama, et al. [9,24]. The identified molecule has a very similar structure to QMPSB with the only difference being due to the presence of two fluorine atoms in position 4’ of the piperidinyl group. Additional results obtained with both monodimensional and bidimensional NMR are presented in the Supplementary Materials: COSY (Figure S11), HSQC-DEPT (Figure S10), HMBC ^15^N-^1^H (Figure S12), HMBC ^13^C-^1^H (Figure S13), TOCSY (Figure S14) and ^19^F NMR (Figure S7). 

### 2.3. Chromatography and HR-MS/MS Results

#### 2.3.1. UHPLC-qTOF-MS

An initial chromatographic screening was performed in order to assess the complexity of the analysed matrix. This assessment is essential, as it can identify possible interferents in the matrix that may affect the reliability of the results. The application of UHPLC-qTOF-MS enables the chromatographic separation of all components of the sample and the subsequent production of the related MS^n^ spectra. As there were no analytical standards available for the substances in question at the time of this work, identification was based on the spectral data followed by the application of cheminformatics tools. The MS and MS/MS data were analysed primarily with the Agilent MassHunter (Agilent Technologies, Santa Clara, CA, USA) and further processed with ACD/Labs Spectrus Processor followed by the MS Fragmenter for structural elucidation and fragmentation prediction confirmation. This software allows the prediction of fragmentation, confronting experimental with theoretical data, and annotates MS and MS/MS spectral ion trees. HR-MS/MS enables the precise determination of the monoisotopic mass and prior knowledge of the theoretical structure from the techniques employed allows the confirmation of the molecule by fragmentation matching.

**Figure 4:**
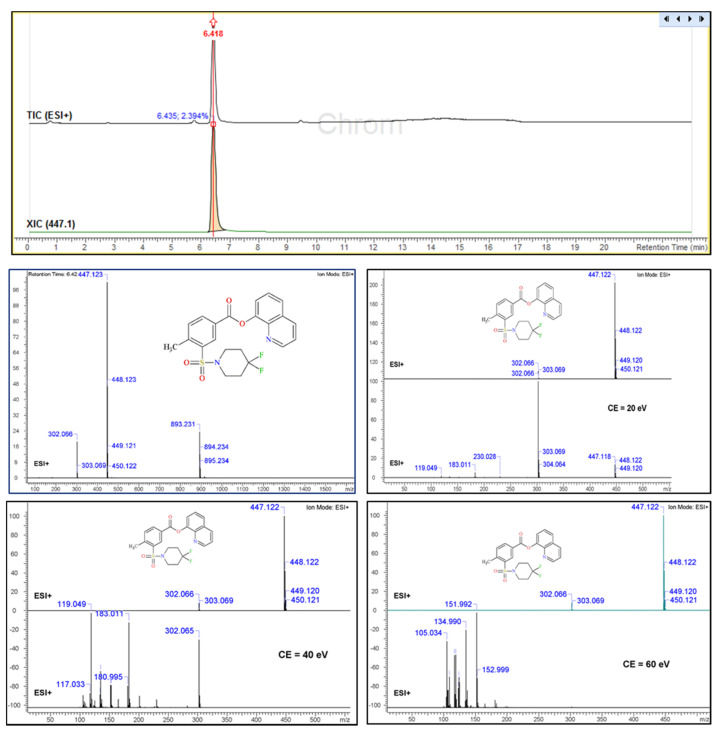
UHPLC-ESI(+) qTOF-MS TIC, MS and MS/MS spectra (20, 40 and 60 eV) of the identified cannabinoid 2F-QMPSB.

It can be seen in Figure 4 that when the sample was analysed with positive ionization it was mainly composed of three substances, one of which was overwhelmingly dominant. The two additional substances showed smaller peaks with *m*/*z* of 429.128 (t_R_ = 5.75 min) and 373.228 (t_R_ = 9.45 min), respectively (Figure 4, Figures S3 and S4). These two peaks represented only 0.5% of the relative content (based on peak areas), and for this reason their presence had a minimal effect on the structural elucidation of the main substance in the sample. 

Regarding the main substance (t_R_ = 6.42 min), the full MS scan showed a base molecular ion with an *m*/*z* of 447.123, in agreement with 2F-QMPSB. 

At this point it should be mentioned that the presence of a fragment with an *m*/*z* of 302.066 was also noted, which was in agreement with that observed in the GC-MS analysis. This fragment seemed to be the 1-carboxy-(methylene-benzenesulfonyl)-4,4-difluoropiperidinyl group (see discussion in Section 3.2) from where the oxyquinoline was detached after the cleavage of the ester bond attached to the oxyquinoline group. After performing the structural confirmation, it was most likely that this product was the result of hydrolysis of the ester bond. The presence of this fragment is most probably due to the dissolution of the extract in a mixture of acetonitrile and water, in combination with the mobile phase, which also consisted of a gradient of acetonitrile and water, in the presence of 0.1% formic acid.

Following the selection of the molecular ion (*m*/*z* = 447.123) an MS/MS fragmentation was performed at three selected collision energies (CID = 20.0, 40.0 and 60.0 eV), to assess the fragmentation pattern (Figure 4). The resulting MS fragments were identified and subsequently confirmed using the ACD/Labs MS fragmenter. 

The fragmentation pattern followed a specific path where the base peak (*m*/*z* 447.123) was broken from the carbonyl ester bond (*m*/*z* 302.066) and also in the sulfonyl group (*m*/*z* 183.011), producing the 4,4-difluoro-1-piperidinyl sulfonyl group ( see discussion in Section 3.2). The increment of the collision energy reflects the generation of various fragments which are reported in Figure 4. The fragmentation pattern of the obtained MS/MS results from the UHPLC-qTOF-MS which confirms the proposed chemical structure. Subsequent data assessment confirmed the findings of GC-IT-MS regarding the fragmentation patterns of 2F-QMPSB and the associated fragmentation pathways are presented and discussed in paragraph 3.2. These results have been further evaluated with those from the flow injection HR-Orbitrap-MS where a difference was observed between the collision energies, regarding the fragmentation of the molecular ion in the MS2 experiments, i.e., in the case of the qTOF-MS (40 eV), which seemed to be higher than those reported with the Orbitrap-MS (25 eV).

**Figure 5:**
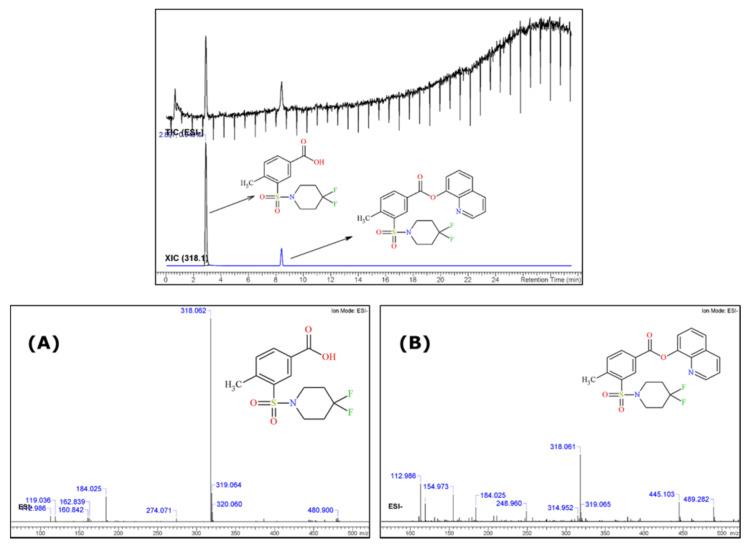
UHPLC-ESI (-) qTOF-MS TIC and ESI(-) MS spectra of the identified precursor 2F-MPSBA (**A**) and of the identified cannabinoid 2F-QMPSB (**B**).

As the GC-MS experiments depicted in Section 2.1 indicated the presence of a 2F-QMPSB precursor substance, a, LC-MS analysis, using negative ionisation (ESI-), was performed (Figure 5). The results confirmed the existence of the precursor 2F-MPSBA in its acid form (*m*/*z* 318.061) in the chloroform extract, which could not be seen using ESI+. The cannabinoid 2F-QMPSB (*m*/*z* 445.103) was also present, but at a much lower abundance when compared with the analysis using positive ionisation.

#### 2.3.2. Flow Injection into Orbitrap MS

**Figure 6:**
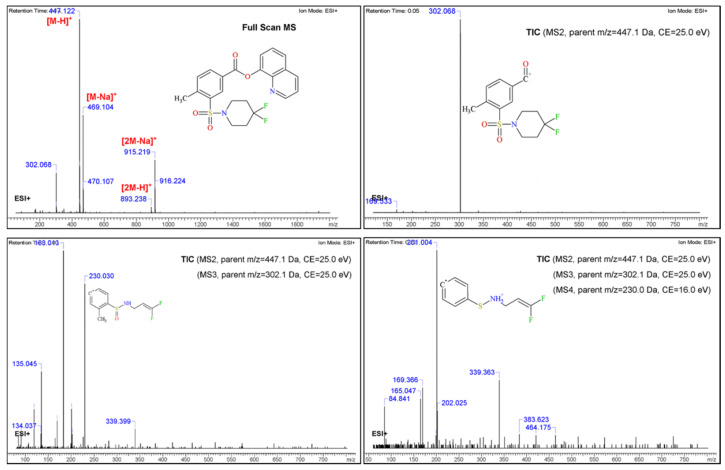
Flow injection Orbitrap MS and MS^E^ spectra of the target analyte 2F-QMPSB.

In this case no hyphenated technique was applied prior to flow injection directly to the Orbitrap, where the MS resolution was 140,000 and the injection flow rate was set at 3 μL/min. The analysis was first performed in the full scan mode, and later on the performance of MS^2^, MS^3^ and MS^4^, sequentially. The resulting MS^E^ spectra, along with the identified fragments, are presented in Figure 6. 

Full scan MS (TIC) was performed initially, where the protonated molecular ion was identified as *m*/*z* 447.122 [M + H]^+^. The TIC results are in full accordance with the results obtained with the qTOF analysis, where the existence of two ions, one at *m*/*z* 302.068 and a less abundant one at *m*/*z* 183.012, could be observed indicating the hydrolysis of the ester bond as well the breakage of the sulfonyl group.

**Figure 7:**
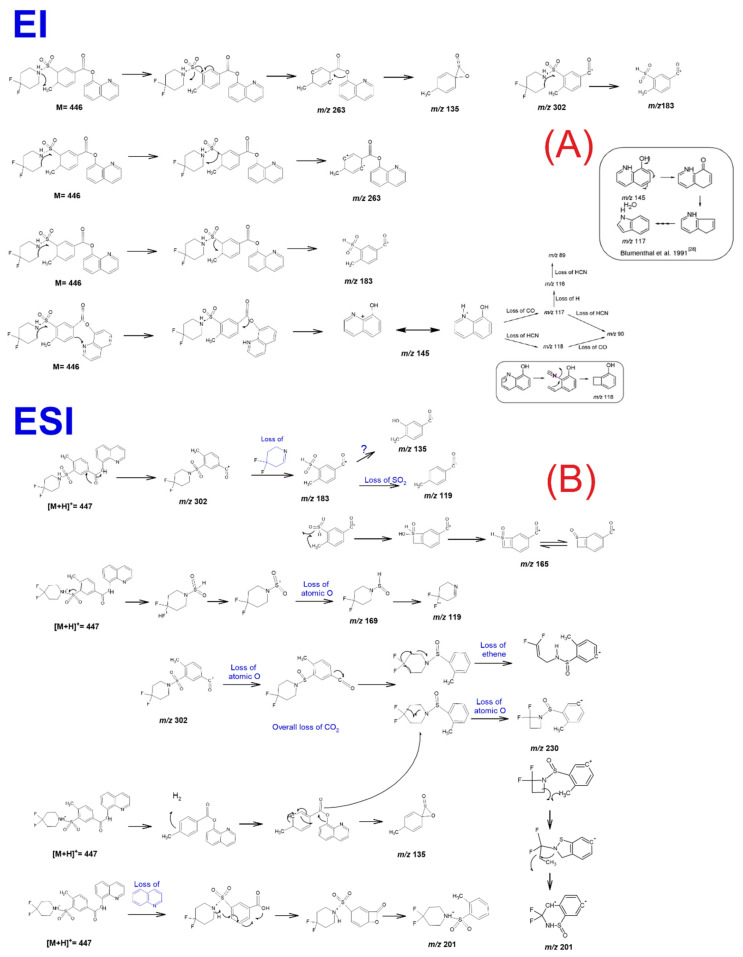
GC-EI-MS (**A**) and UHPLC-ESI HR-MS (**B**) suggested fragmentation schemes for identified NPS 2F-QMPSB.

For the MS^2^, the aforementioned molecular ion (*m*/*z* 447.120) was selected to be fragmented, while for the MS^3^ the ions *m*/*z* 302.068 and *m*/*z* 230.030, representing the fragment (Figure 7B) with the open piperidine structure, were further investigated in the MS^4^ experiment. The fragmentation pattern, collision energies and MS^E^ identified fragments are in accordance with the UHPLC-qTOF-MS experiments and with the proposed chemical structure. In addition, the evaluation of the Orbitrap-MS results demonstrated the alignment between the initial findings with GC-IT-MS and those of the qTOF-MS, regarding the spectral data of the NPS 2F-QMPSB.

## 3. Discussion

### 3.1. NMR

The ^1^H and ^13^C chemical shifts and *J* coupling constants, measured in DMSO-d_6_ (Table 1), are in accordance with those found by Blakey, et al., and Uchiyama, et al. [9,24], for a molecule that is very similar in structure to QMPSB, The only difference observed between these two molecules was due to the presence of two fluorine atoms which were observed in the piperidinyl group, as shown in Figure 3.

Similarly, considering the chemical shift data listed in Table 1, this difference can be seen in the ^13^C-NMR spectrum for the carbon atom at position 4’ (Figure 3), where the two hydrogen atoms are substituted by fluorine atoms. In the ^1^H-NMR spectrum, the difference is found mainly in the protons linked to the carbons at positions 2’,6’ and 3’,5’ of the piperidinyl group.

Furthermore, the coupling constants (Table 1) are also in accordance with those reported [9] apart from those which were generated by the coupling with the two fluorine atoms bonded to the quaternary carbon 4’. This is clearly revealed in the ^13^C spectrum where coupling (^13^C and ^19^F have spins of ½) gives a triplet with a ^1^*J*_CF_ coupling constant of 241.9 Hz. We observed a similar splitting phenomenon of the carbon signals 3’,5’ (t, ^2^*J*_CF_ = 23.7 Hz) and 2’,6’ (t, ^3^*J*_CF_ = 5.5 Hz).

The *J*_CF_ coupling constants are in line with those found in the literature, in particular with those of Jeffries, et al. and Doddrell, et al. [25,26]. The latter presented the behaviour of 1,1-difluorocyclohexane, provided that the chair–chair interconversion could be halted. The molecular dynamics of 1,1-difluorocyclohexane, where the *J*_CF_ coupling constants are ^1^*J*_CF_ = 242 Hz the ^2^*J*_CF_ = 24.0 Hz and ^3^*J*_CF_ = 4.7 Hz, assigned to C-1 (attached to the two fluorine atoms), C-2, and C-3, respectively [26]. Jeffries, et al. presented the synthesis of molecules bearing the 4,4-difluoropiperidine scaffold and NMR data of the 4,4-difluoropiperidine moiety which are consistent with those presented in this paper [25] as well as being in accordance with studies carried out by Yousif and Roberts [27], and Edzes, et al. and Furuya, et al. for gem-difluorides [28,29].

The monodimensional fluorine spectrum and the bidimensional spectra, confirm the proposed structure and are presented in the Supplementary Materials (Table S2 and Figures S5–S14). The NMR spectra measured in CDCl_3_ confirm that which was observed in the DMSO-d_6_ spectra. However, the signals in the aromatic region in CDCl_3_ are less well separated than in DMSO-d_6_, whereas those in the aliphatic region, in particular for the ^1^H signals of 2’,6’ and b2’,b6’, are better defined as they are not overlapped by the water signal (Figures S5 and S6).

### 3.2. Fragmentation Patterns by EI+ and ESI+

Figure 7 presents the suggested fragmentation scheme, independent of the applied EI+ or ESI+ MS detection, for all the identified and confirmed fragments of 2F-QMPSB.

The additional reported substance in the GC-MS analysis was found to be the gem-fluorine substituted MMPSB (2F-MMPSB; Figure 1A,B), obtained as the methyl ester derivative of its acid form 2F-MPSBA after extraction from the herbal material matrix with methanol. When chloroform was used for extraction from the same material, the peak of the precursor molecule is not present, as no transesterification with methanol occurs during extraction. The precursor remains in its acid form, which is not observable on a DB5-MS analytical column (Figure 1B). Blakey, et al. [9], reported a similar *m*/*z* of 149 for MMPSB [4-methyl-3-(1-piperidinylsul-fonyl) benzoate]. The *m*/*z* 121 fragment was also observed, which refers to the fragmented 4,4-difluoropiperidine coming from the cleavage of the methylated 2F-MPSBA and is also consistent with the GC-MS results reported by Blakey, et al. [9]. The existence of this precursor compound was further confirmed by UHPLC-qTOF-MS analysis, where it could be identified but only in the ESI-mode (acid form). All identification results of the unknown peak in GC-MS and qTOF-MS, have been checked and confirmed by ACD/Labs data platform.

Regarding the MS of the newly identified cannabinoid, the base fragment was at *m*/*z* 302, representing the acylium ion arising from the cleavage of the 8-hydroxyquinoline group (Figure 7A) [30]. Another identified fragment was at *m*/*z* 183, representing the 4,4-difluoro–1-piperidinylsulfonyl unit (Figure 7A). In addition, certain structurally rearranged peaks were identified and subsequently assessed and evaluated with the UHPLC-qTOF-MS and the Orbitrap MS.

In the LC-qTOF-MS (ESI+) TIC analysis of the chloroform extract only three peaks were observed, as shown in Figure 4, Figures S3 and S4. The most abundant one (around 98%, based on the area normalization approach) corresponded to 2F-QMPSB. The two remaining peaks were evaluated using the cheminformatics platform, with the first one having an *m*/*z* of 429.128 and possibly representing the monofluorinated QMPSB (Figure S3). It can be speculated that this was formed either due to the removal of fluorine from the piperidine group or due to a left-over compound arising from the chemical synthesis of the target molecule. The second peak presented a *m*/*z* of 373.228 (Figure S4), which after processing of the spectrum with ACD/Labs was tentatively identified as a possible trace of the synthetic cannabinoid MAM-2201, as reported by Blakey, et al. [9].

An interesting observation, coming mainly from the qTOF-MS and Orbitrap-MS fragmentation results (not so clear in the GC-MS results), was the formation of an intermediate arylsulfinamide (*m*/*z* 169) from the main arylsulfonamide (*m*/*z* 183). This is due to the polarization of the C-S bonds, which appears to be very important for SO_2_ rearrangement, as previously reported, where during ionisation the positive charge of the protonated molecule most likely resides on the S=O group [31,32].

The formation of arylsulfinamide, or the elimination of the SO_2_, could be enhanced by neighboring group participation, such as the N-S (Figure 7B). It should also be highlighted that the SO_2_ rearrangement pathway, in the positive mode, is less predictable [31,32].

### 3.3. Final Considerations on the 2F-QMPSB

The work described in this manuscript represents the process involved in the first chemical identification of 2F-QMPSB, in seized samples, which led to its reporting to the EU Early Warning System [23]. At the moment of this first identification, a search for that chemical structure was performed which led only to a record found only in the PiHKAL isomer-design Website, where it was presented with the common name of ‘SGT 13’ [33]. Discussions about SGT-13 were found on social media where its correct IUPAC chemical name was cited and its possible effects outlined. This was found interesting because this substance does not belong to the typical category of the synthetic cannabinoids based on the indole or indazole core group, which are the more popular among drug users. After further investigation regarding the possible origin of this name, we also found a patent by Bowden and Williamson, related to a series of indole and indazole cannabinoid compounds [34]. However, SGT-13 and its corresponding chemical structure were not described in this patent. On the other hand, another patent from 2007 on novel sulfamoyl benzamide compounds, proposed that they may act as as agonists or antagonists of the cannabinoid receptor system [35]. This was experimentally confirmed by studies of some such compounds and in particular QMPSB [12,13]. However, it seems that until now only a few detections of these compounds were found in seized materials of recreational drugs. A recent publication reports the recent emergence of such sulfamoyl benzoate, sulfamoyl benzamide, and *N*-benzoylpiperidine based structures [36].

## 4. Materials and Methods

### 4.1. Chemicals and Reagents

All solvents used for LC-HR-MS/MS analysis, obtained from Sigma-Aldrich (Milan, Italy) were LC-MS Chromasolv grade. Ultrapure water (18.2 MΩ) was obtained from a Milli-Q system (Millipore, Burlington, MA, USA). Deuterated dimethylsulfoxide (DMSO-d6) and deuterated chloroform (CDCl_3_) were >99.8% deuterated and were also obtained from Sigma-Aldrich.

A parcel, destined for an Italian address, was seized in February 2018 from a postal shipment coming from Spain, by the Customs Officials and the Finance Guard of Malpensa Airport in Italy. Its contents were declared as “environment perfumers/ herbal incense”, and it was made up of nine unlabeled transparent plastic vials containing an oily straw-yellow liquid and eight plastic bags of herbal material. All samples were analyzed by GC-IT-MS at the customs laboratory in Milan, which identified several known synthetic cannabinoids in the methanol extract of the oily liquid samples and in five of these the herbal material samples. For three of the unlabeled plastic bags (samples 7, 8, and 9 of the present work), the GC-IT-MS analyses showed various concentrations of the same unkown substance with no match in the mass spectrometry libraries. Aliquots of these three samples were sent for further analysis to the JRC.

### 4.2. Sample Preparation

For the GC-IT-MS analysis, approximately 300–500 mg of the herbal material was placed in a glass tube with 10 mL of methanol, sonicated for 15 min and filtered with a PTFE 0.22 μm filter. An aliquot was then injected in the GC-MS system. 

For the analysis performed at the JRC, a similar extraction procedure was employed, but with 3 mL of deuterated chloroform (CDCl_3_) as the solvent. An amount of 650 μL of the filtrate was then analysed directly by NMR. Another 650 μL aliquot was left to evaporate to dryness and then redissolved with 650 μL of deuterated DMSO-d_6_ for other NMR analysis with this solvent.

For HR-MS/MS, a portion of the CDCl_3_ extracts was first diluted 1:5 *v*/*v* with acetonitrile, and then further diluted with a solution of 1:1 *v*/*v* acetonitrile: H_2_O. The final sample had a concentration of around 2 mg/mL for both the LC and the direct infusion HR-Orbitrap-MS analysis.

### 4.3. Instrumental Analysis

The methods and analytical techniques are already described in several previous works and in references therein [6,21,37]. 

#### 4.3.1. GC-MS

A Varian 4000 Gas Chromatograph (Varian, Palo Alto, CA, USA) equipped with an Ion Trap Mass Detector (GC-IT-MS) was used in the Italian Customs Laboratory (Milan). The GC column used was a DB5-MS from Agilent Technologies (30 m, 0.250 mm, 0.25 mm) with helium as the carrier gas (1 mL/min). The temperature program used was as follows: 2 min isothermal at 220 °C, ramp at 8 °C/min up to 300 °C and isothermic for 15 min (total run time 27 min). An injection volume of 1 µL in split mode (80:1) and injector at 290 °C have been used. Scan mode was used ranging from *m*/*z* 40 to 450. 

At the JRC, an Agilent 7890, equipped with a 5975C MSD detector, was used with the same settings and conditions of analysis as described above for the Italian customs laboratory.

#### 4.3.2. NMR

Spectra were acquired on a Bruker (Rheinstetten, Germany) spectrometer Avance III HD 600 (nominal proton frequency 600.13 MHz), equipped with a 5 mm QCI cryo-probe (^1^H, ^13^C, ^15^N and ^19^F), in DMSO-d_6_ and CDCl_3_ solvent at 295 K and 300 K, respectively. ^1^H and ^13^C NMR chemical shifts are expressed in δ scale (ppm) and referenced to the solvent residuals, at 2.50 ppm and 39.52 ppm respectively for DMSO-d_6_ or 7.23 ppm and 77.0 ppm for CDCl_3_.

#### 4.3.3. HR-MS/MS

- UHPLC-qTOF-MS analysis

A UHPLC system (Agilent 1290) with a qTOF-MS (Agilent 6540 UHD Accurate-Mass, Agilent, Waldbronn, Germany), with ESI+ (4 kV) and ESI- (−3 kV) ionization modes, were used. The source temperature was 325 °C. Nitrogen was used as both the drying (40 psi) and nebulizing gas (10 L min-1). The injection volume was 5 μL. The TOF-MS detector was set for a *m*/*z* range of 100–1600, with acquisition of MS/MS high resolution accurate mass data. The fragmentation collision energies for the ESI (+) ranged from 5–60 eV.

A Waters (Waters, Milford, MA, USA) BEH C18 (100 × 2.1 mm, 1.7 μm) analytical column was used at 40 °C. The mobile phase (flow rate: 200 μL min^−1^) consisted of water (A) and methanol (B), both with 0.1% formic acid. The gradient program changed linearly from 50% to 95% (B) in 25 min, followed by an isocratic elution for 4 min and an equilibration time of 1 min to reach initial mobile phase conditions.

- Direct infusion to Orbitrap-MS

Thermo LTQ Orbitrap MS (Thermo Scientific, Bremen, Germany). Analysis was performed with ESI+ operated with mass resolution of 140,000 at *m*/*z* 400. The chloroform extracts of the herbal material were first diluted in pure acetonitrile, followed by another dilution with H_2_O to have a final 1:1 *v*/*v* ratio. The resulting samples were then infused at a flow rate of 5 μL/min on the system.

### 4.4. Cheminformatics 

The ACD/Labs suite (ACD/Labs, Toronto, ON, Canada) was used, together with MassHunter (Agilent Technologies) and XCalibur (Thermo Scientific) to assess and evaluate the relevant data for structural elucidation of the substances under investigation.

## 5. Conclusions

New synthetic cannabimimetic substances are reaching the European illicit drugs market and posing considerable challenges to both customs and control laboratories regarding their identification and subsequent control. This underlines the need for new and systematic analytical approaches such as the one outlined in this study.

The identification of NPS remains challenging and although GC-MS is a powerful detection tool, additional techniques, such as those detailed here, are required for the absolute analytical confirmation of new substances. In the present work, the combination of HR-MS, NMR and cheminformatics techniques proved effective in the structural elucidation of the new synthetic cannabinoid quinolin-8-yl-3-[(4,4-difluoropiperidin-1-yl) sulfonyl]-4-methylbenzoate (2F-QMPSB), the identification of which is reported here for the first time. It was also possible to identify the presence of an acid precursor of 2F-QMPSB, the 4-methyl-3-(4,4-difluoro-1-piperidinylsulfonyl) benzoic acid (2F-MPSBA). 

## Figures and Tables

**Table 1:** ^1^H and ^13^C NMR peak assignments and coupling constants for 2F-QMPSB.

Carbon	δ^13^C/ppm	J_FC_	δ ^1^H/ppm	J_HH_
C = O	163.5			
1	127.3			
2	130.7		8.60	d, *J* = 1.65 Hz
3	136.6			
4	144.0			
CH3	20.3		2.71	s
5	134.2		7.78	d, *J* = 7.9 Hz
6	134.3		8.40	dd, *J* = 7.9, 1.65 Hz
2’,6’	42.3	t, *J* = 5.5 Hz	3.34	t, *J* = 5.3 Hz
3’,5’	33.2	t, *J* = 23.2 Hz	2.09	m
4’	121.9	t, *J* = 241 Hz		
2’’	150.8		8.87	dd, *J* = 4.1, 1.4 Hz
3’’	122.3		7.62	dd, *J* = 8.3, 4.1 Hz
4’’	136.4		8.50	dd, *J* = 8.3, 1.4 Hz
4a’’	129.2			
5’’	126.6		8.00	dd, *J* = 8.1, 1.1 Hz
6’’	126.5		7.70	dd, *J* = 8.1, 7.3 Hz
7’’	121.8		7.75	dd, *J* = 7.3, 1.1 Hz
8’’	146.8			
8a’’	140.2			

## Data Availability

Electronic data can be available upon request to the corresponding authors.

## Supplementary Materials

The supplementary materials are available online.

## Sample Availability

Samples of the compounds are not available from the authors.
