# Supplementary material for: Identification and Analytical Characterization of a Novel Synthetic Cannabinoid-Type Substance in Herbal Material in Europe
Source: Molecules. 2021 Feb 3;26(4):793. doi: 10.3390/molecules26040793 (PMC7913736; doi:10.3390/molecules26040793)
Supplement: Supplementary file 1 [file molecules-26-00793-s001.pdf]

Identification and analytical characterization of a novel synthetic cannabinoid-type substance in herbal material in Europe

Emmanouil D. Tsochatzis[1,2], Joao A. Lopes [1, *], Margaret V. Holland[1], Fabiano Reniero[1], Giovanni Palmieri[3] and Claude Guillou [1, *]

[1] European Commission, Joint Research Centre, Via E. Fermi, 2749, TP 281, I-21020 Ispra (VA), Italy;, Emmanouil.TSOCHATZIS@ec.europa.eu , Margaret.HOLLAND@ec.europa.eu, Fabiano.RENIERO@ec.europa.eu
[2] present address: Department of Food Science, Aarhus University, Agro Food Park 48, 8200, Aarhus N, Denmark;
[3] Agenzia Dogane Monopoli, Direzione Regionale per la Lombardia, Laboratorio e Servizi Chimici, 20138, Milan, Italy; giovanni.palmieri01@agenziadogane.it
* Correspondence: Joao-Filipe.ALBERTO-LOPES@ec.europa.eu; Claude.GUILLOU@ec.europa.eu; +390332785678 (C.G)

SUPPLEMENTARY MATERIAL

Figure S1 : Chemoidentifiers and IUPAC name of 2F-QMPSB

Figure S2 : Chemoidentifiers and IUPAC name of 2F-MPSBA

Table S1 : Fragments of the two compounds identified by GC-IT-MS

Figure S3 : UHPLC-qTOF-MS, minor compound 1 at t_R=5.75 min (monofluorinated QMPSB)

Figure S4 : UHPLC-qTOF-MS, minor compound 2 at t_R=9.45 min (AM-2201)

Figure S5 : ^{1}H NMR spectrum of Sample 8 in $CDCl_3$

Figure S6 : Comparison of ^{1}H NMR spectra in CDl_3 of the three samples (zoom regions of aromatic and aliphatic signals)

Figure S7 : ^{19}F spectrum in $CDCl_3$

Figure S8 : ^{1}H NMR spectrum of Sample 8 in DMSO-d6

Figure S9 : Comparison of ^{1}H NMR spectra in DMSO-d6 of the three samples (zoom regions of aromatic and aliphatic signals)

Table S2 : ACD/labs NMR summary table (Sample 8)

Figure S10 : 2D-NMR HSQC in DMSO-d6 (Sample 8)

Figure S11 : 2D-NMR COSY in DMSO-d6 (Sample 8)

Figure S12 : 2D-NMR HMBC ^{15}N, ^{1}H in DMSO-d6 (Sample 8)

Figure S13 : 2D-NMR HMBC ^{13}C, ^{1}H in DMSO-d6 (Sample 8)

Figure S14 : 2D-NMR TOCSY ^{1}H, ^{1}H in DMSO-d6 (Sample 8)

Note: *Electronic data can be obtained upon request to the authors.*

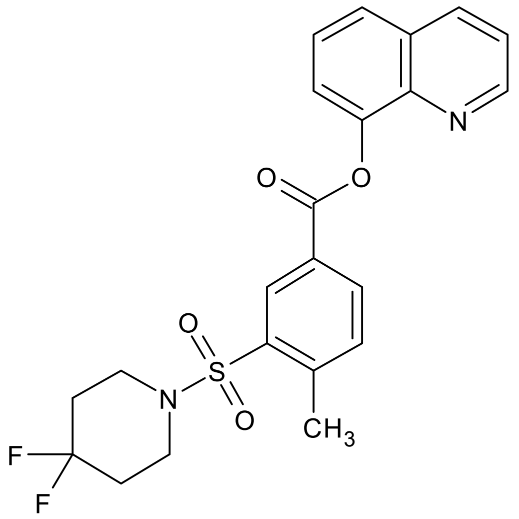

Molecular Formula: $C_{22}H_{20}F_2N_2O_4S$
Formula Weight: 446.4670064
Monoisotopic Mass: 446.111184 Da
Nominal Mass: 446 Da
Average Mass: 446.467 Da

quinolin-8-yl 3-(4,4-difluoropiperidine-1-sulfonyl)-4-methylbenzoate

InChI=1S/C22H20F2N2O4S/c1-15-7-8-17(14-19(15)31(28,29)26-12-9-22(23,24)10-13-26)21(27)30-18-6-2-4-16-5-3-11-25-20(16)18/h2-8,11,14H,9-10,12-13H2,1H3

InChiKey: JOSWCKYCXJMLNM-UHFFFAOYSA-N

SMILES: FC1(F)CCN(CC1)S(=O)(=O)c1cc(ccc1C)C(=O)Oc1cccc2cccnc21

Figure S1 : Chemoidentifiers and IUPAC name of 2F-QMPSB

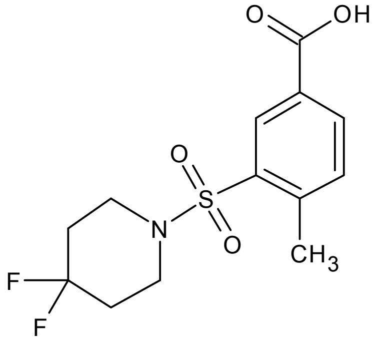

Molecular Formula: $C_{13}H_{15}F_2NO_4S$
Formula Weight: 319.3243064
Monoisotopic Mass: 319.068984 Da
Nominal Mass: 319 Da
Average Mass: 319.3243 Da

3-(4,4-difluoropiperidine-1-sulfonyl)-4-methylbenzoic acid

InChI=1S/C13H15F2NO4S/c1-9-2-3-10(12(17)18)8-11(9)21(19,20)16-6-4-13(14,15)5-7-16/h2-3,8H,4-7H2,1H3,(H,17,18)

InChiKey: VRCWMNIBLGREDC-UHFFFAOYSA-N

SMILES: FC1(F)CCN(CC1)S(=O)(=O)c1cc(ccc1C)C(=O)O

Figure S2 : Chemoidentifiers and IUPAC name of 2F-MPSBA

Table S1. Fragments of the two compounds identified by GC-IT-MS

1st peak t_R = 5.38 min						
**No.**	**Formula**	**Fragment**	**m/z Calc**	**m/z Exp.**	**RI Exp.(%)**	**TIC Exp.(%)**
1	C14H17F2NO4S(+)	M	333.084	333	9.713	2.289
2	C13H14F2NO3S(+)	M - CH3O	302.066	302.1	5.863	1.362
3	C12H13FNO2S(+)	M - C2H4FO2	254.065	254.1	8.45	1.926
4	C9H10O2(+)	M - C5H7F2NO2S	150.068	150.1	9.527	2.001
5	C9H9O2(+)	M - C5H8F2NO2S	149.06	149.1	21.646	4.545
6	C9H8O2(+)	M - C5H9F2NO2S	148.052	148.1	18.189	3.819
7	C8H6O2(+)	M - C6H11F2NO2S	134.036	134.1	6.716	1.394
8	C5H9F2N(+)	M - C9H8O4S	121.07	121.1	23.869	4.791
9	C5H8F2N(+)	M - C9H9O4S	120.062	120.1	100	20.069
10	C5H7F2N(+)	M - C9H10O4S	119.054	119.2	17.513	3.514
11	C5H6F2N(+)	M - C9H11O4S	118.046	118.2	10.607	2.128
12	C5H5F2N(+)	M - C9H12O4S	117.038	117.2	19.572	3.927
13	C5H7FN(+)	M - C9H10FO4S	100.056	100.1	13.504	2.71
14	C4H5F2(+)	M - C10H12NO4S	91.035	91.1	14.374	2.842
15	C7H6(+)	M - C7H11F2NO4S	90.046	90.1	16.578	3.388
16	C7H5(+)	M - C7H12F2NO4S	89.039	89.1	32.206	6.582
**2nd-peak t_R = 17.54 min ; 2F-QMPSB**						
**No.**	**Formula**	**Fragment**	**m/z Calc**	**m/z Exp.**	**RI Exp.(%)**	**TIC Exp.(%)**
1	C13H15F2NO3S(+)	M - C9H5NO	303.074	303.1	12.405	1.403
2	C13H14F2NO3S(+)	M - C9H6NO	302.066	302.1	100	11.307
3	C13H13FNO3S(+)	M - C9H7FNO	282.059	282.1	10.651	1.204
4	C13H12FNO3S(+)	M - C9H8FNO	281.052	281.1	26.457	2.991
5	C17H13NO2(+)	M - C5H7F2NO2S	263.094	263.2	59.35	6.651
6	C15H10NO2(+)	M - C7H10F2NO2S	236.071	236.1	6.326	0.693
7	C7H9F2NO2S(+)	M - C15H11NO2	209.032	209	14.662	1.547
8	C5H7F2NO2S(+)	M - C17H13NO2	183.016	183	27.875	2.877
9	C9H7NO(+)	M - C13H13F2NO3S	145.052	145.1	7.155	0.732
10	C8H7O2(+)	M - C14H13F2N2O2S	135.044	135.1	25.457	2.572
11	C8H6O2(+)	M - C14H14F2N2O2S	134.036	134.1	6.944	0.701
12	C8H5O2(+)	M - C14H15F2N2O2S	133.028	133.1	6.678	0.675
13	C5H7F2N(+)	M - C17H13NO4S	119.054	119.1	18.368	1.793
14	C5H6F2N(+)	M - C17H14NO4S	118.046	118.1	37.785	3.689
15	C5H5F2N(+)	M - C17H15NO4S	117.038	117.1	14.133	1.38
16	C8H4O(+)	M - C14H16F2N2O3S	116.026	116.1	17.799	1.793
17	C5H7F2(+)	M - C17H13N2O4S	105.051	105.1	5.835	0.568
18	C4H5F2(+)	M - C18H15N2O4S	91.035	91.1	13.439	1.293
19	C7H6(+)	M - C15H14F2N2O4S	90.046	90	12.351	1.228
20	C7H5(+)	M - C15H15F2N2O4S	89.039	89	20.054	1.994

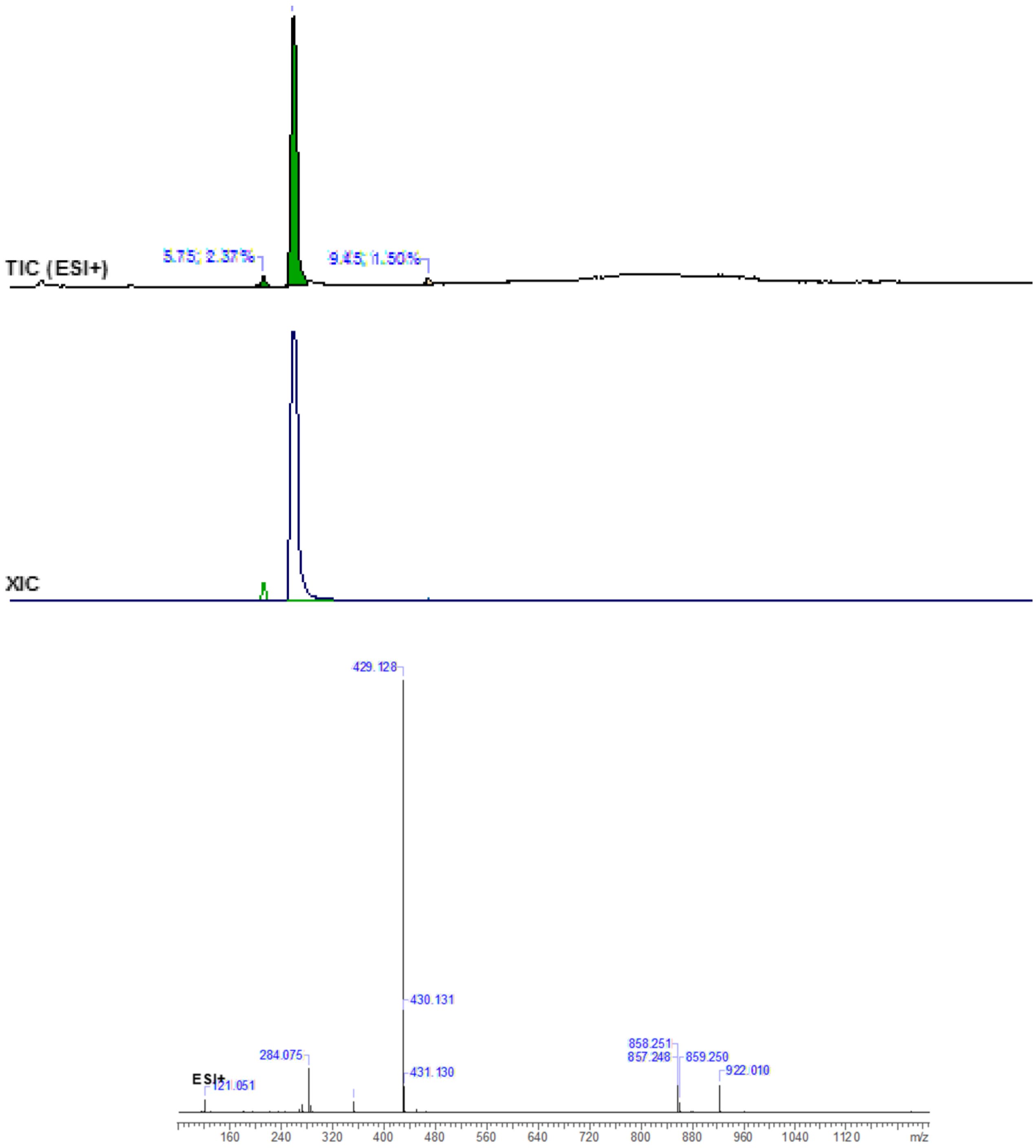

Figure S3 : UHPLC-qTOF-MS, minor compound 1 at t_R = 5.75 min (monofluorinated QMPSB)

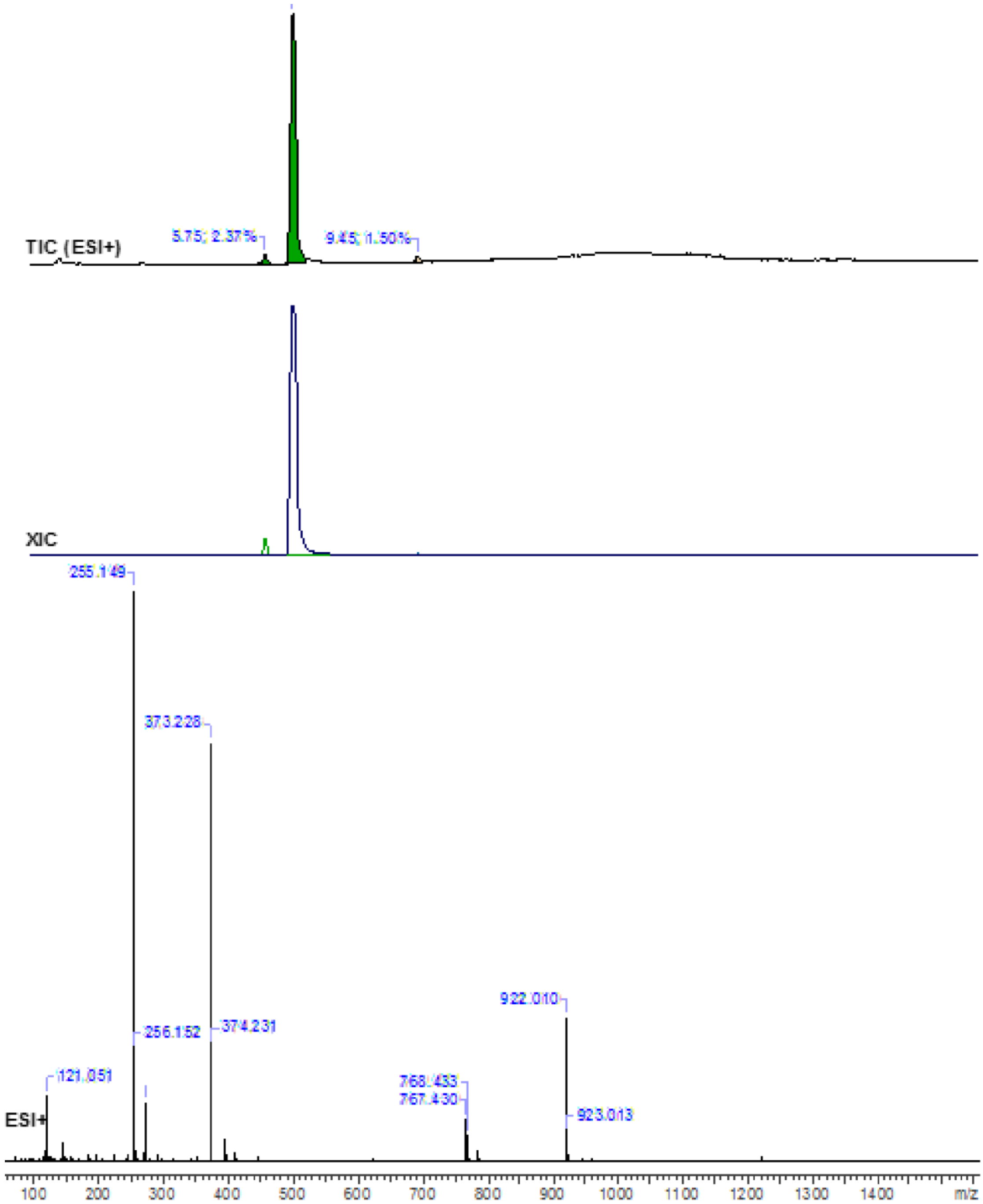

Figure S4 : UHPLC-qTOF-MS, minor compound 2 at t_R=9.45 min (AM-2201)

Sample 8

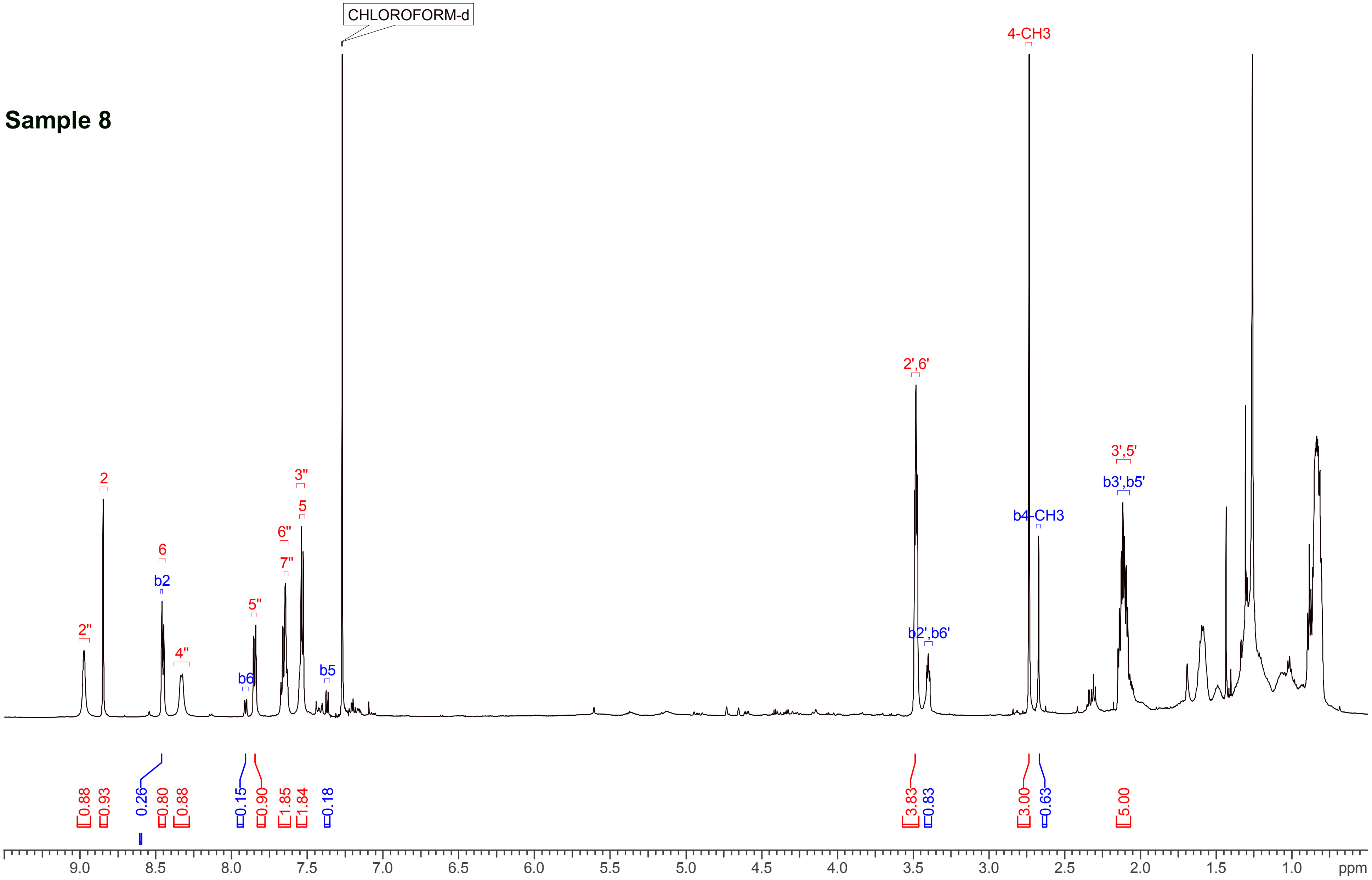

Figure S5 : ^{1}H NMR spectrum of Sample 8 in $CDCl_3$

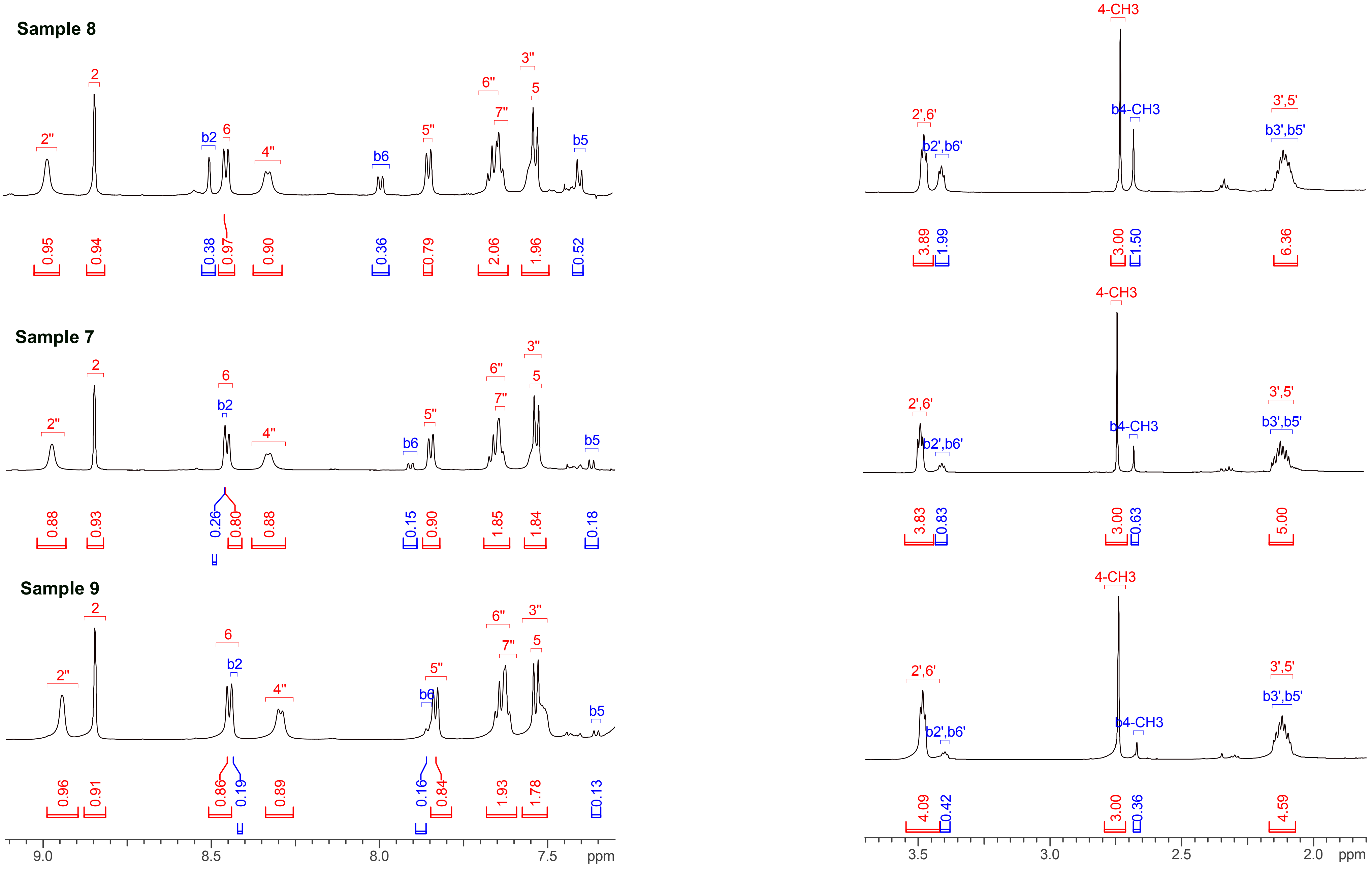

Figure S6 : Comparison of ^{1}H NMR spectra in $CDCl_3$ of the three samples (zooms on aromatic and aliphatic areas)

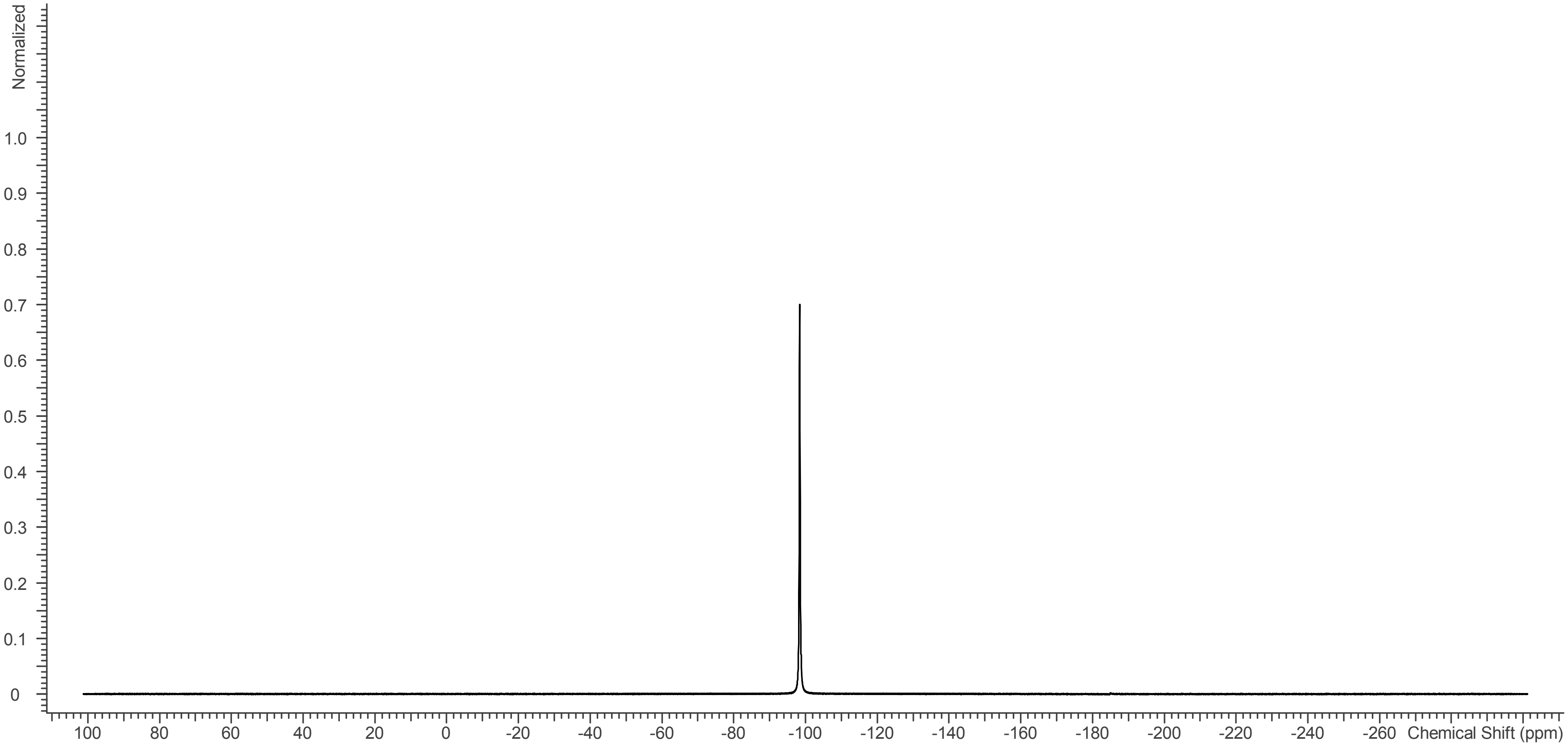

Figure S7 : ^{19}F spectrum in $CDCl_3$ (measured at 295 K , Inverse Gate Decoupling from ^{1}H)

Sample 8

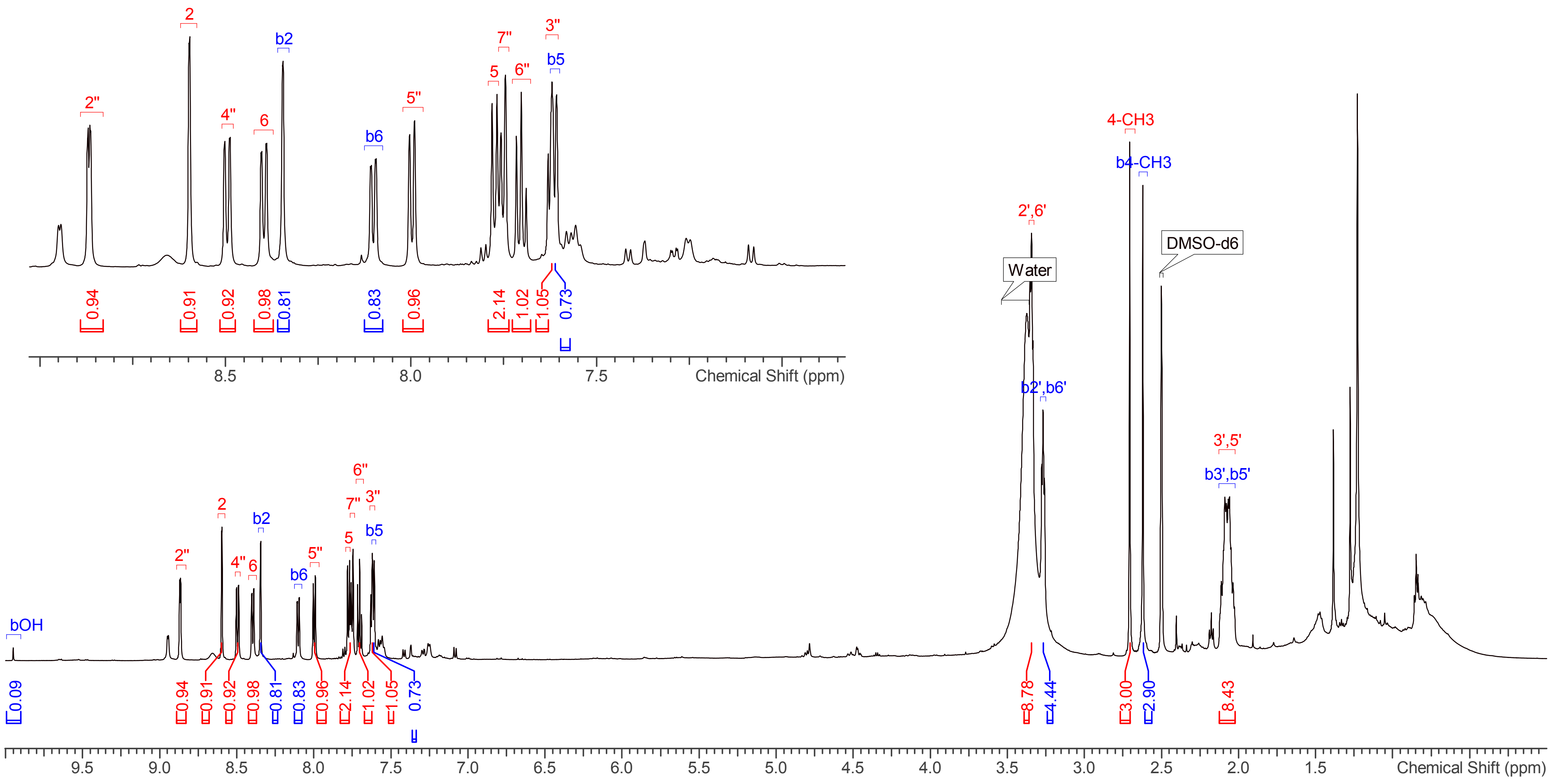

Figure S8 : ^{1}H NMR spectrum of Sample 8 in DMSO-d6

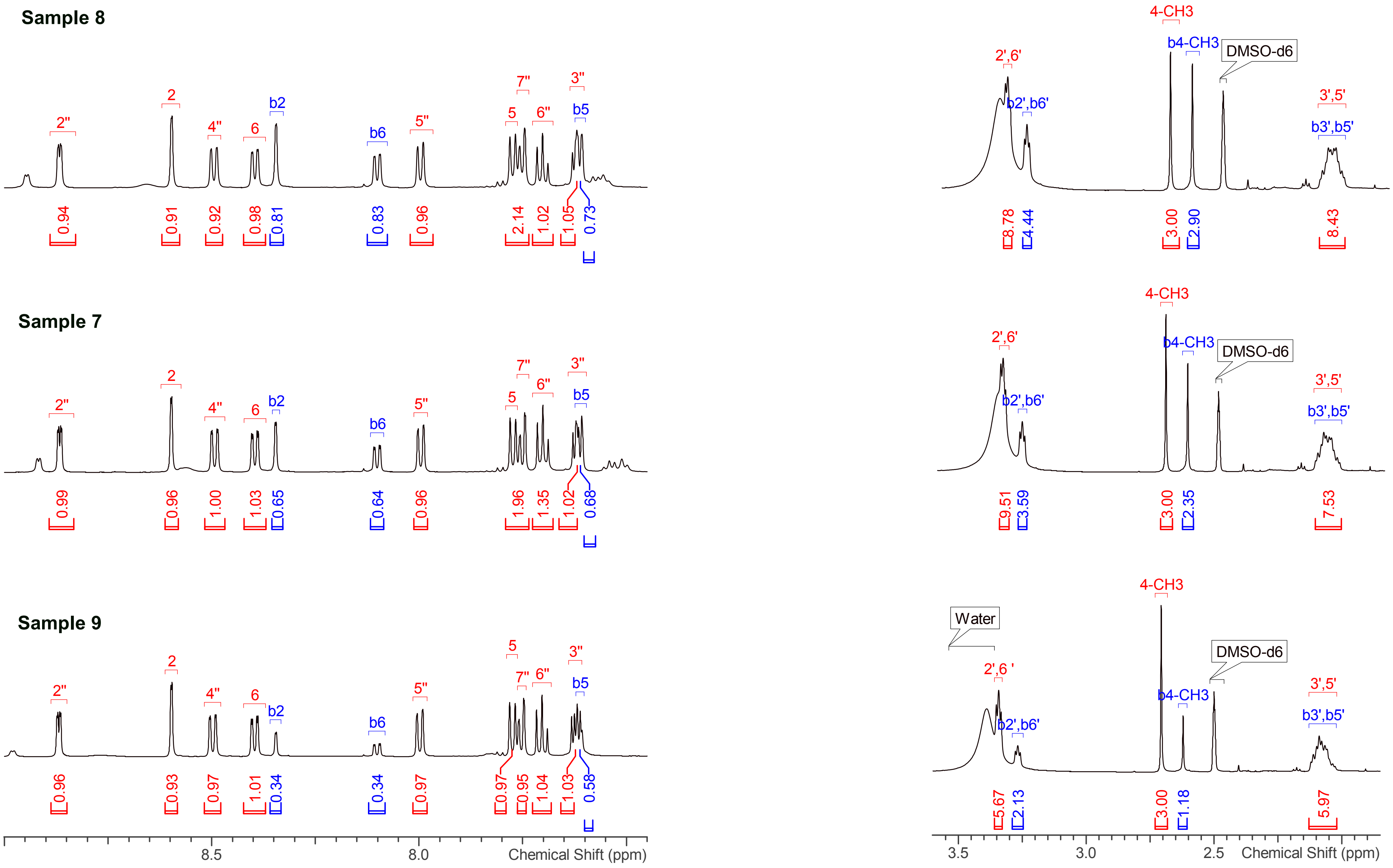

Figure S9 : Comparison of ^{1}H NMR spectra in DMSO-d6 of the three samples (zooms on aromatic and aliphatic areas)

Table S2 : ACD/labs NMR summary table (Sample 8)

The columns C Label and H Label give the correspondence with the convention of numbering of atoms of QMPSB as in Blakey *et al*, 2016.

COSY

TOCSY

HMBC

2F-MPSBA

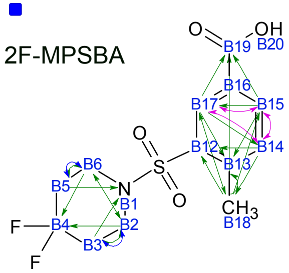

2F-QMPSB

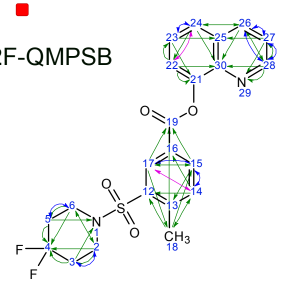

Atom#	C Label	C Shift	X Shift	XHn	H Label	H Shift	H Multiplicity	COSY	TOCSY	H HMBC	C HMBC	X HMBC	Component
B20				OH	bOH	9.950	s						B
B19	C=O	165.984		C						B18, B15, B17			B
B18	b4-CH3	20.157		CH3	b4-CH3	2.621	s			B14, B17	B17, B15, B12, B13, B19		B
B17	b2	130.220		CH	b2	8.345	d (1.56)		B14, B15	B18, B15	B18, B14, B13, B19		B
B15	b6	133.664		CH	b6	8.101	dd (7.89, 1.28)		B14, B17	B18	B17, B13, B19		B
B14	b5	133.702		CH	b5	7.611	br d (7.56)		B15, B17	B17	B18, B12		B
B13	b4	142.508		C						B18, B15, B17			B
B12	b3	135.994		C						B18, B14			B
B4	b4'	121.900		C						B2, B6			B
B3, B5	b3',b5'	33.116		CH2	b3',b5'	2.055	m	B2, B6	B2, B6	B2, B6	B2, B6	B1	B
B2, B6	b2',b6'	42.319		CH2	b2',b6'	3.268	t (5.73, 5.73)	B3, B5	B3, B5	B3, B5	B3, B5, B4		B
B1	bN		92.700	N						B3, B5			B
30	8a''	140.251		C						22, 24, 26, 28			A
29	N2		300.740	N						27, 28			A
28	2''	150.846		CH	2''	8.867	dd (4.10, 1.40)	27, 26	27, 26	27, 26	27, 26, 30	29	A
27	3''	122.294		CH	3''	7.620	dd (8.30, 4.10)	26, 28	26, 28	28	25, 28	29	A
26	4'''	136.399		CH	4''	8.494	dd (8.30, 1.40)	27, 28	27, 28	24, 28	24, 30, 28		A
25	4a''	129.194		C						27, 23			A
24	5''	126.556		CH	5''	7.996	br dd (8.10, 1.10)	23	22	26	22, 26, 30		A
23	6''	126.505		CH	6''	7.702	br dd (8.10, 7.30)	24		22	25, 21		A
22	7''	121.801		CH	7''	7.751	dd (7.30, 1.10)		24	24	23, 30, 21		A
21	8''	146.809		C						23, 22			A
19	C=O	163.456		C						18, 15, 17			A
18	4-CH3	20.303		CH3	4-CH3	2.706	s			14, 17	14, 15, 12, 19		A
17	2	130.684		CH	2	8.597	d (1.65)	15	14, 15	15	18, 15, 19		A
16	1	127.333		C						14			A
15	6	134.311		CH	6	8.396	dd (7.90, 1.65)	14, 17	14, 17	18, 17	17, 13, 19		A
14	5	134.191		CH	5	7.774	d (7.90)	15	15, 17	18	18, 16, 12		A
13	4	143.959		C						15			A
12	3	136.631		C						18, 14			A
4	4'	121.920		C						3, 5, 2, 6			A
3, 5	3',5'	33.172		CH2	3',5'	2.078	m	2, 6	2, 6	2, 6	4	1	A
2, 6	2',6'	42.324		CH2	2',6'	3.343	t (5.30, 5.30)	3, 5	3, 5		3, 5, 4		A
1	N1		92.802	N						3, 5			A

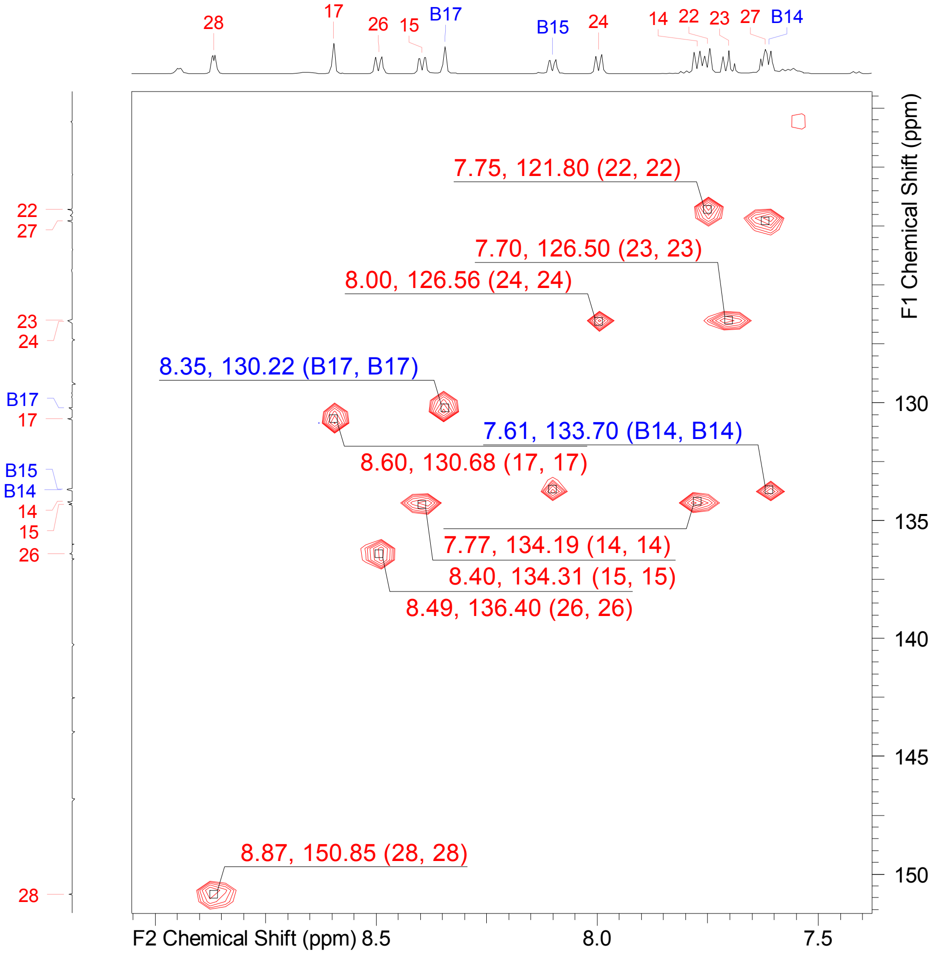

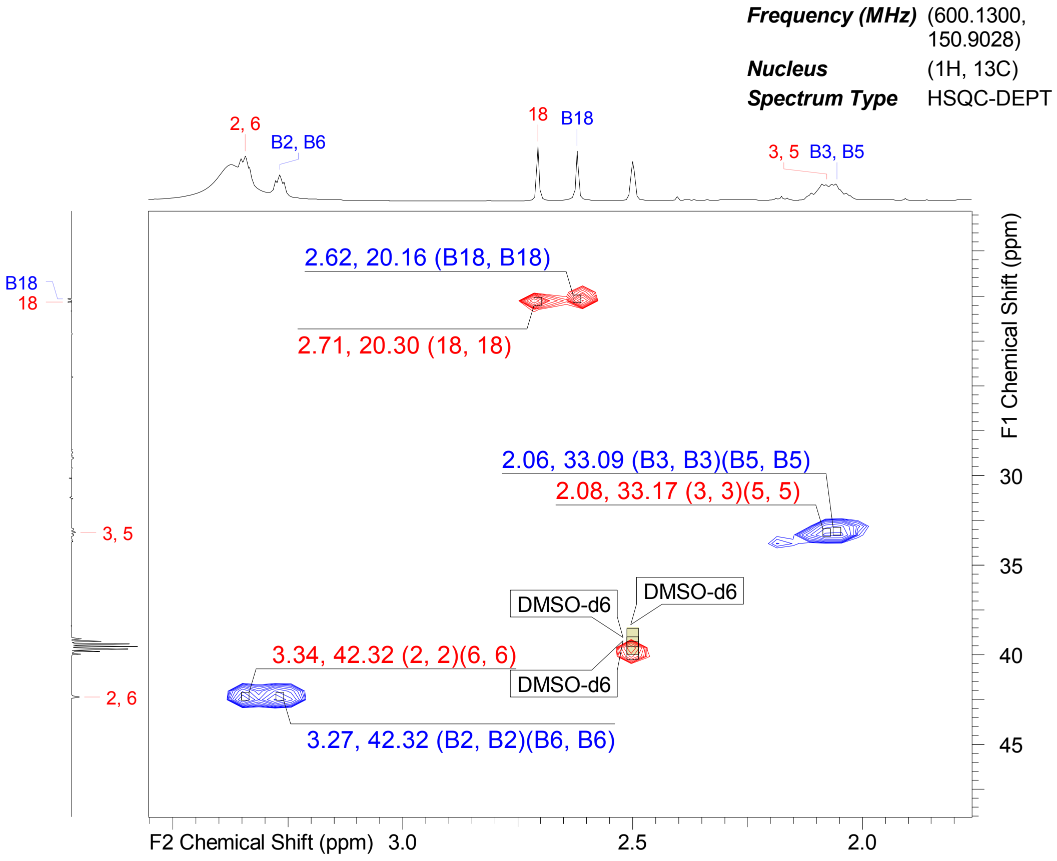

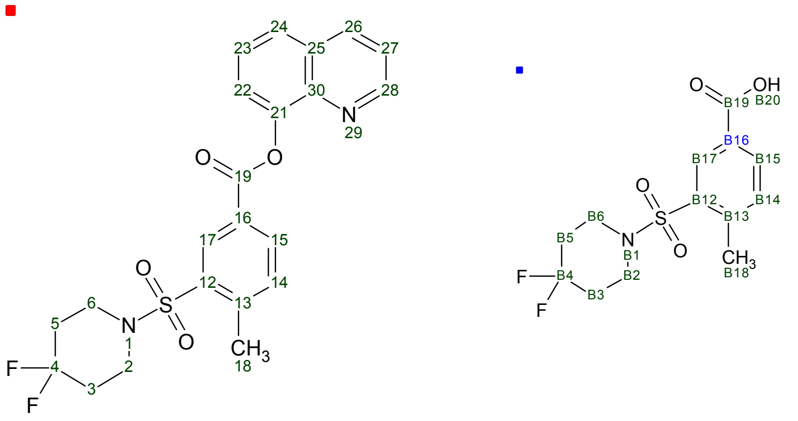

Figure S10 : 2D-NMR HSQC in DMSO-d6 (Sample 8)

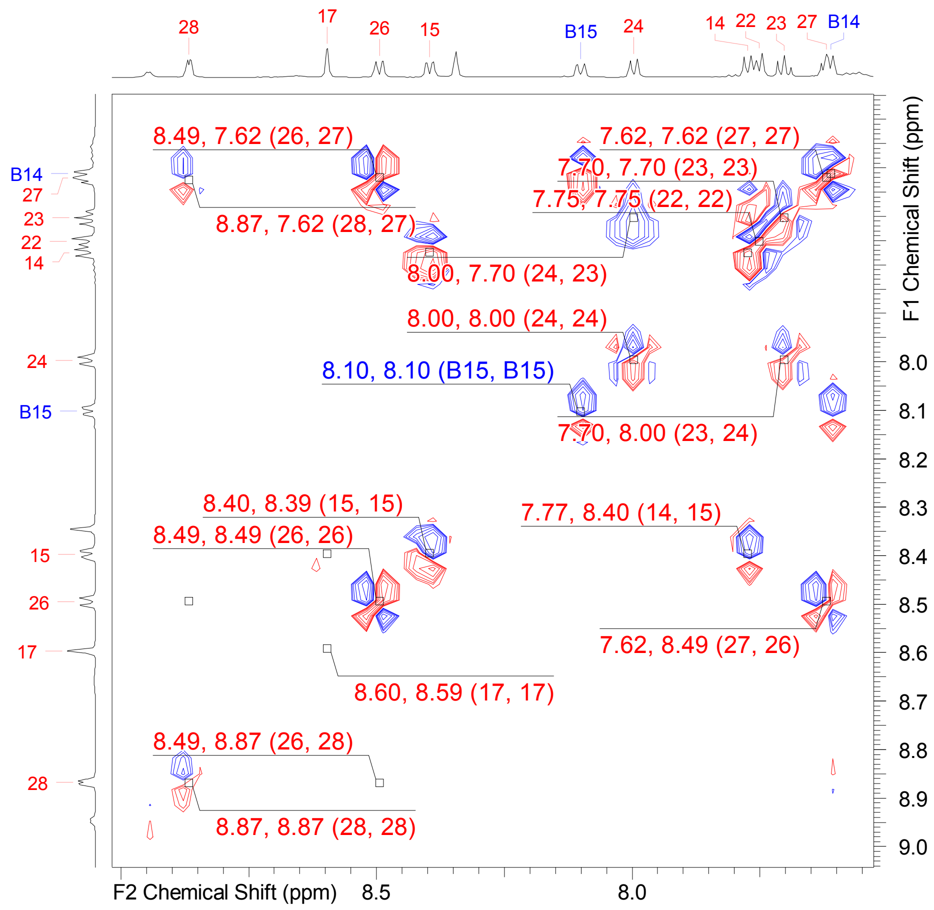

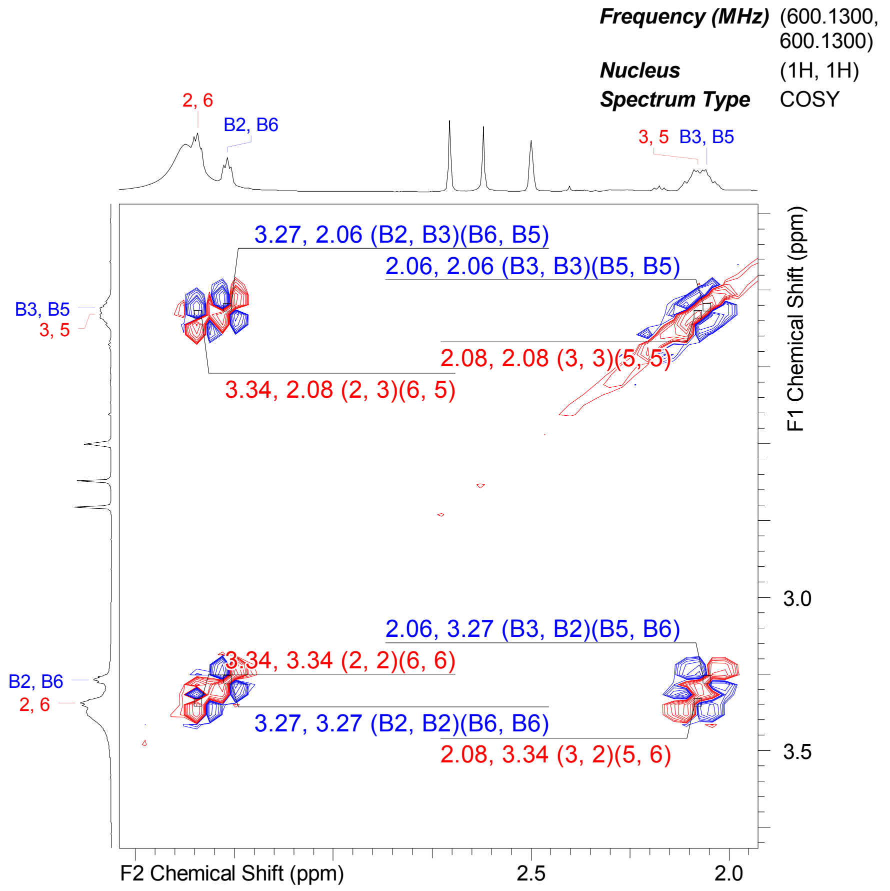

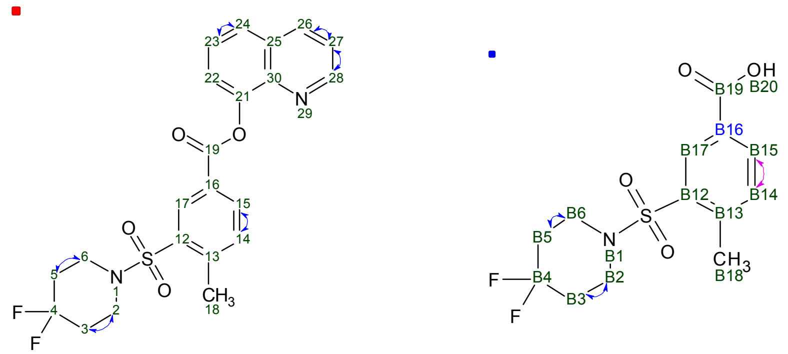

Figure S11 : 2D-NMR COSY in DMSO-d6 (Sample 8)

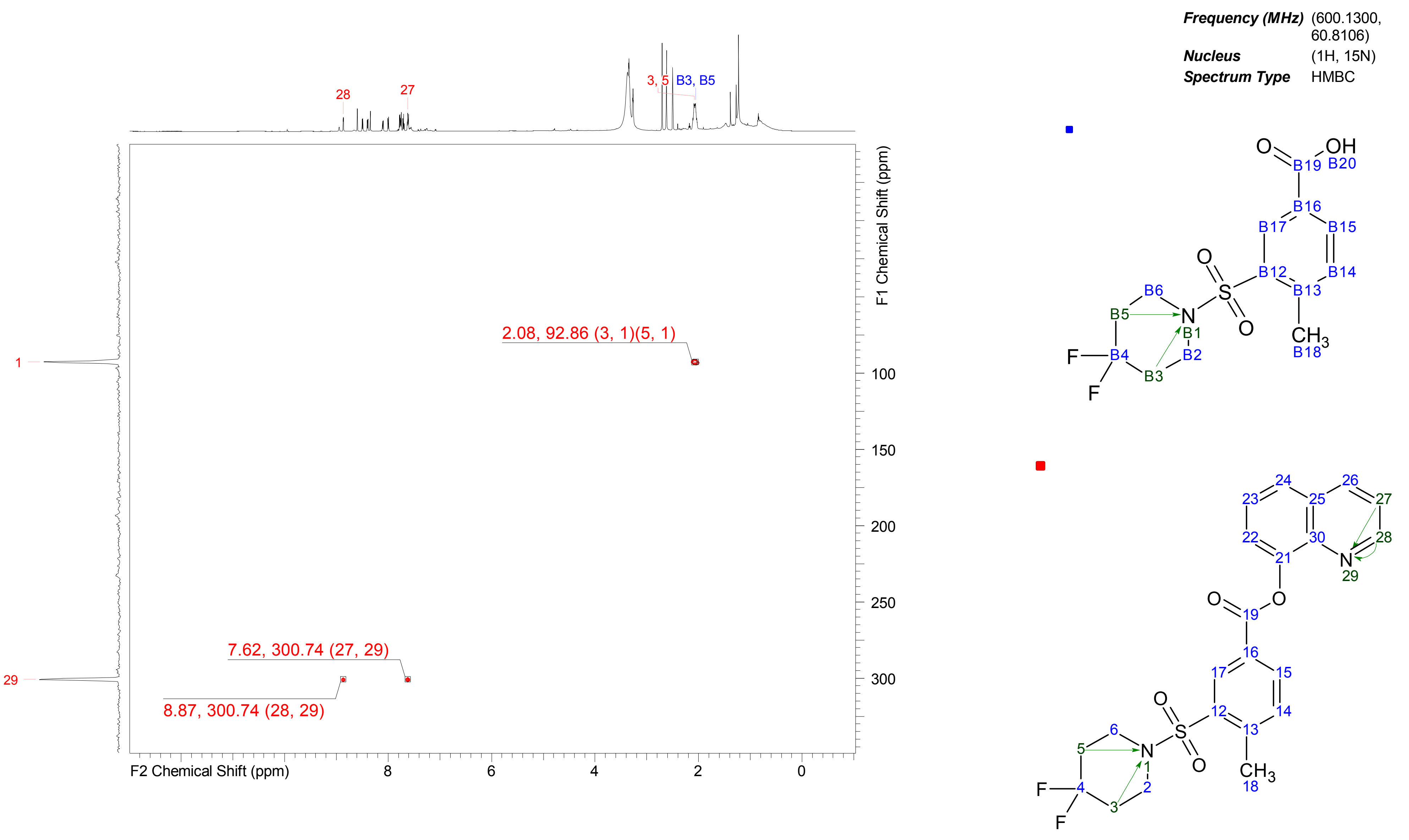

Figure S12 : 2D-NMR HMBC ^{15}N, ^{1}H in DMSO-d6 (Sample 8)

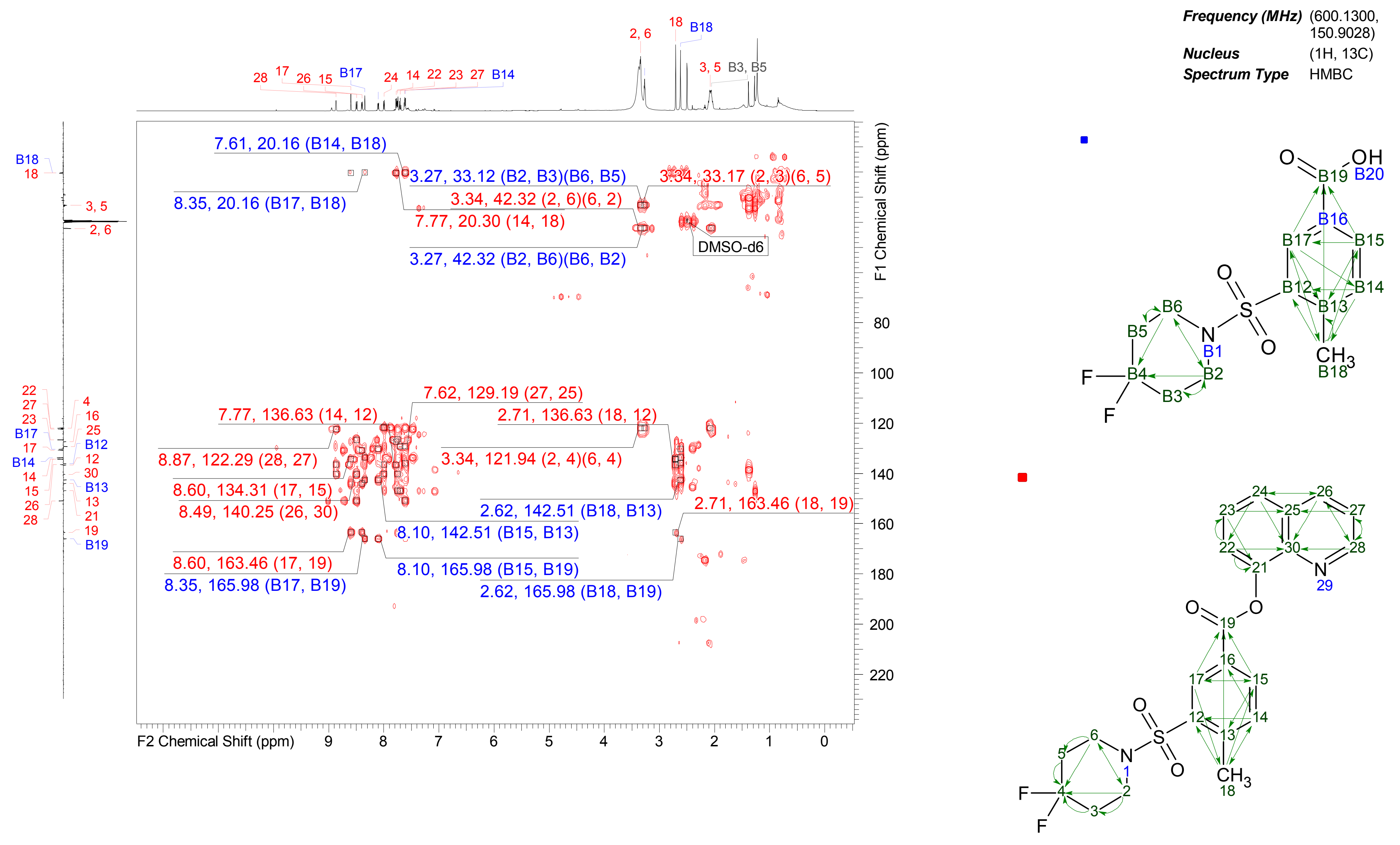

Figure S13 : 2D-NMR HMBC ^{13}C, ^{1}H in DMSO-d6 (Sample 8)

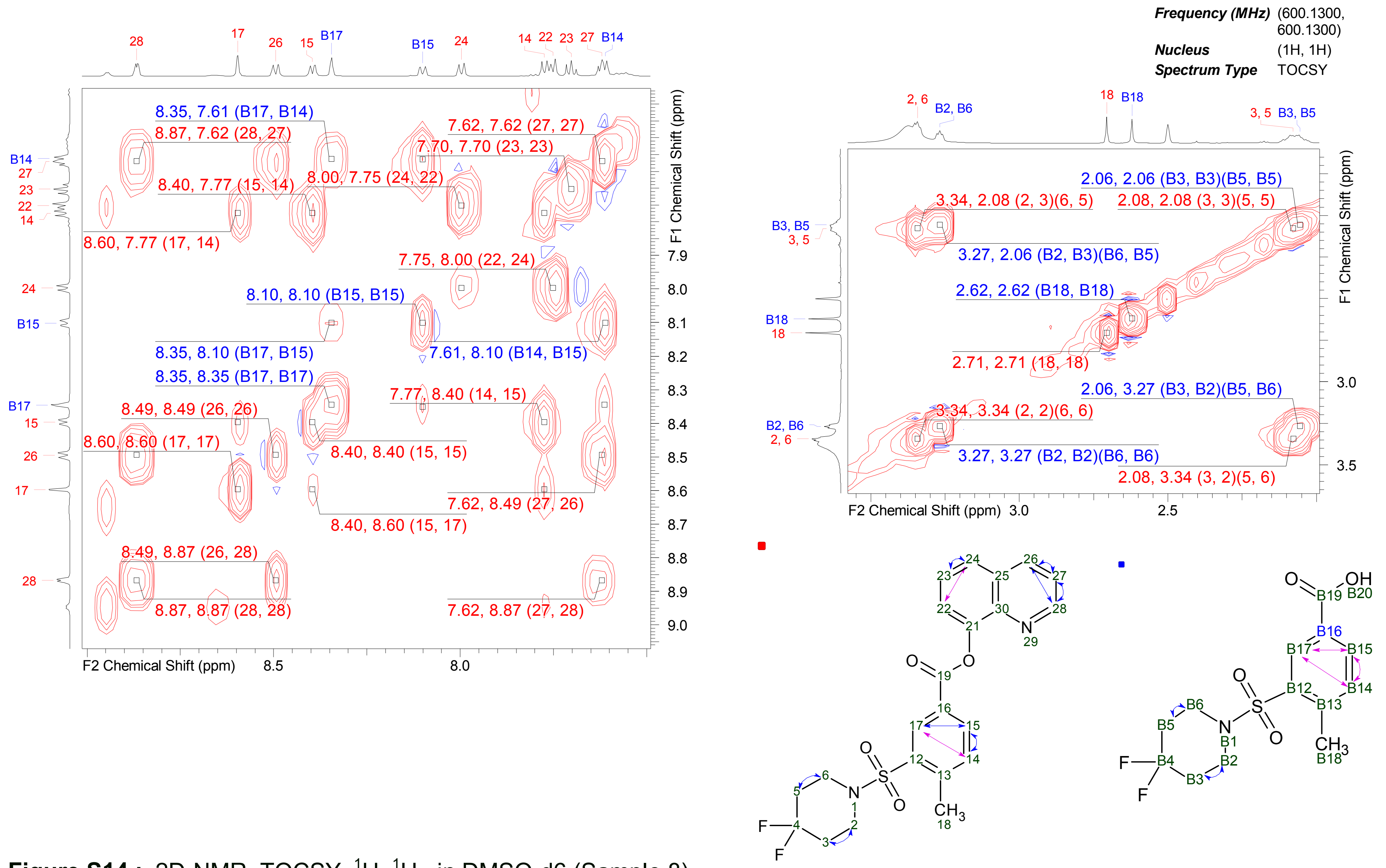

Figure S14 : 2D-NMR TOCSY ^{1}H, ^{1}H in DMSO-d6 (Sample 8)
